# Supplementary material for: Detecting cell-of-origin and cancer-specific methylation features of cell-free DNA from Nanopore sequencing
Source: Genome Biol. 2022 Jul 15;23:158. doi: 10.1186/s13059-022-02710-1 (PMC9283844; doi:10.1186/s13059-022-02710-1)
Supplement: Supplementary file 1 — Additional file 1. Supplementary Figs. S1-S10. [file 13059_2022_2710_MOESM1_ESM.pdf]

Fig S1

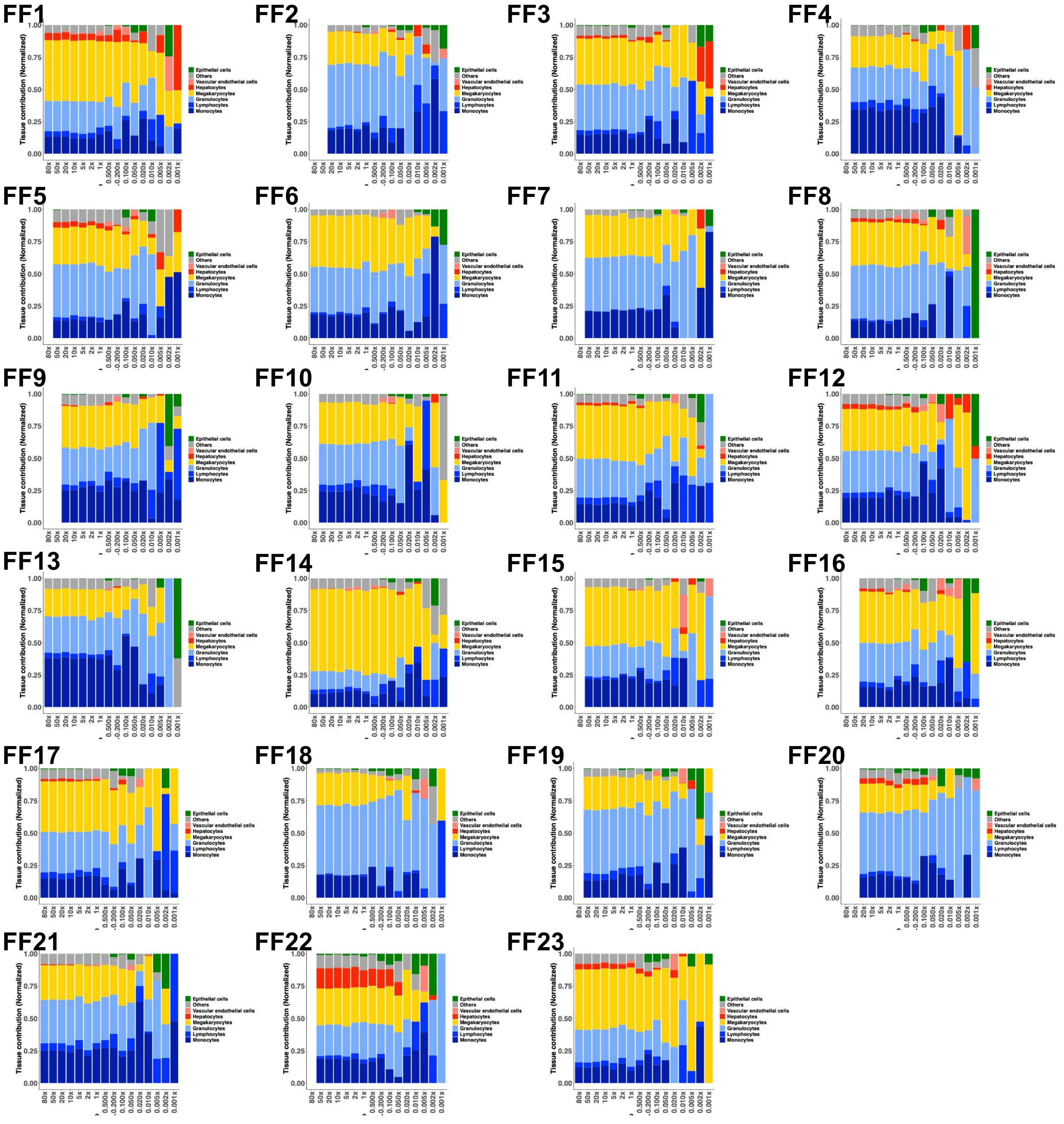

Figure S1: DNA methylation deconvolution for Fox-Fisher et al. samples. Each sample from [1] was downsampled from full depth to 0.001x coverage, and sample ordering is the same as Fig 1B-C. Short names are used, and full sample information is available in Supplementary Table 2.

Fig S2

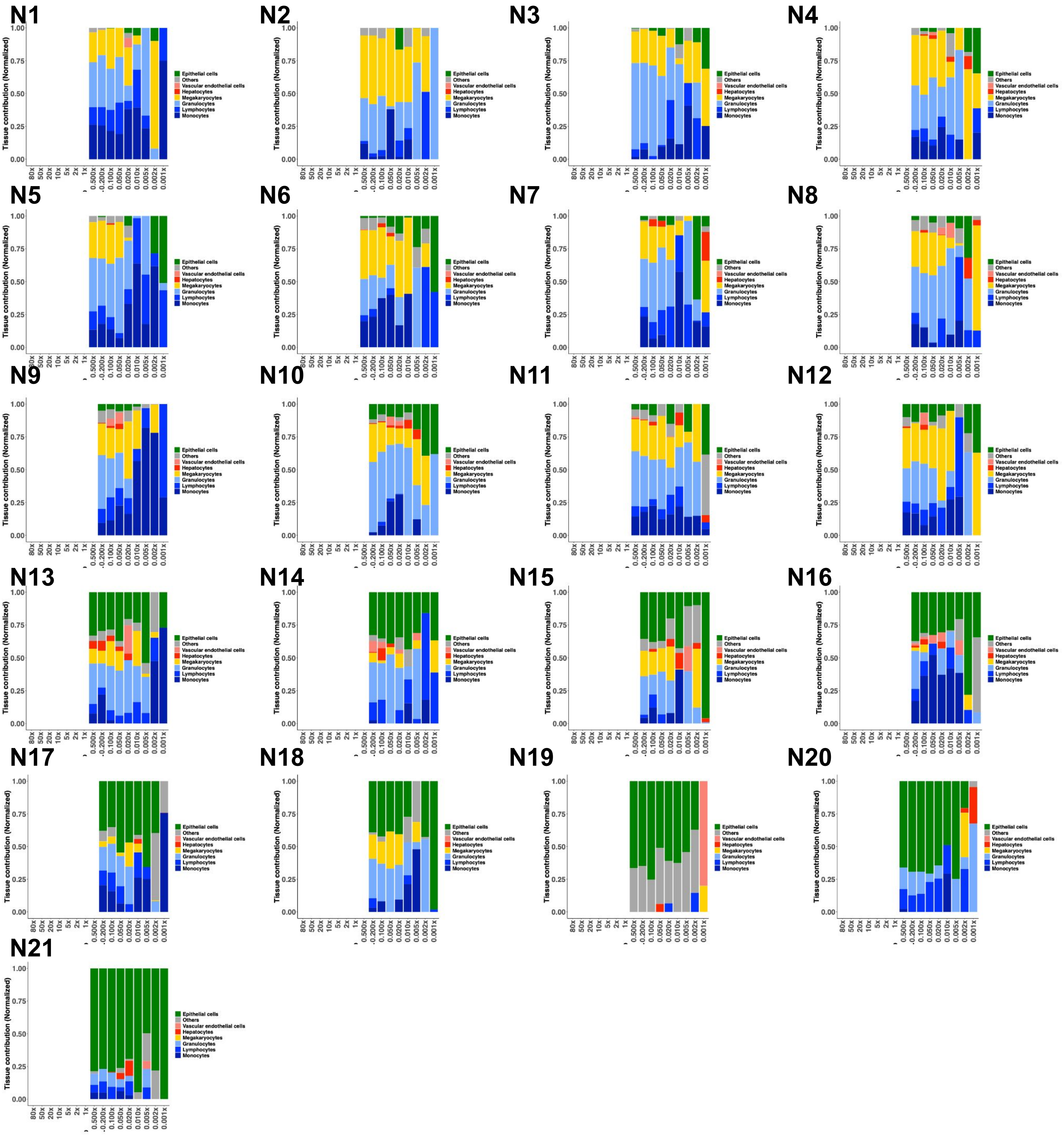

Figure S2: DNA methylation deconvolution for Nguyen et al. samples. Each sample from [2] was downsampled from full depth to 0.001x coverage, and sample ordering is the same as Fig 1B-C. Short names are used, and full sample information is available in Supplementary Table 2.

Fig S3

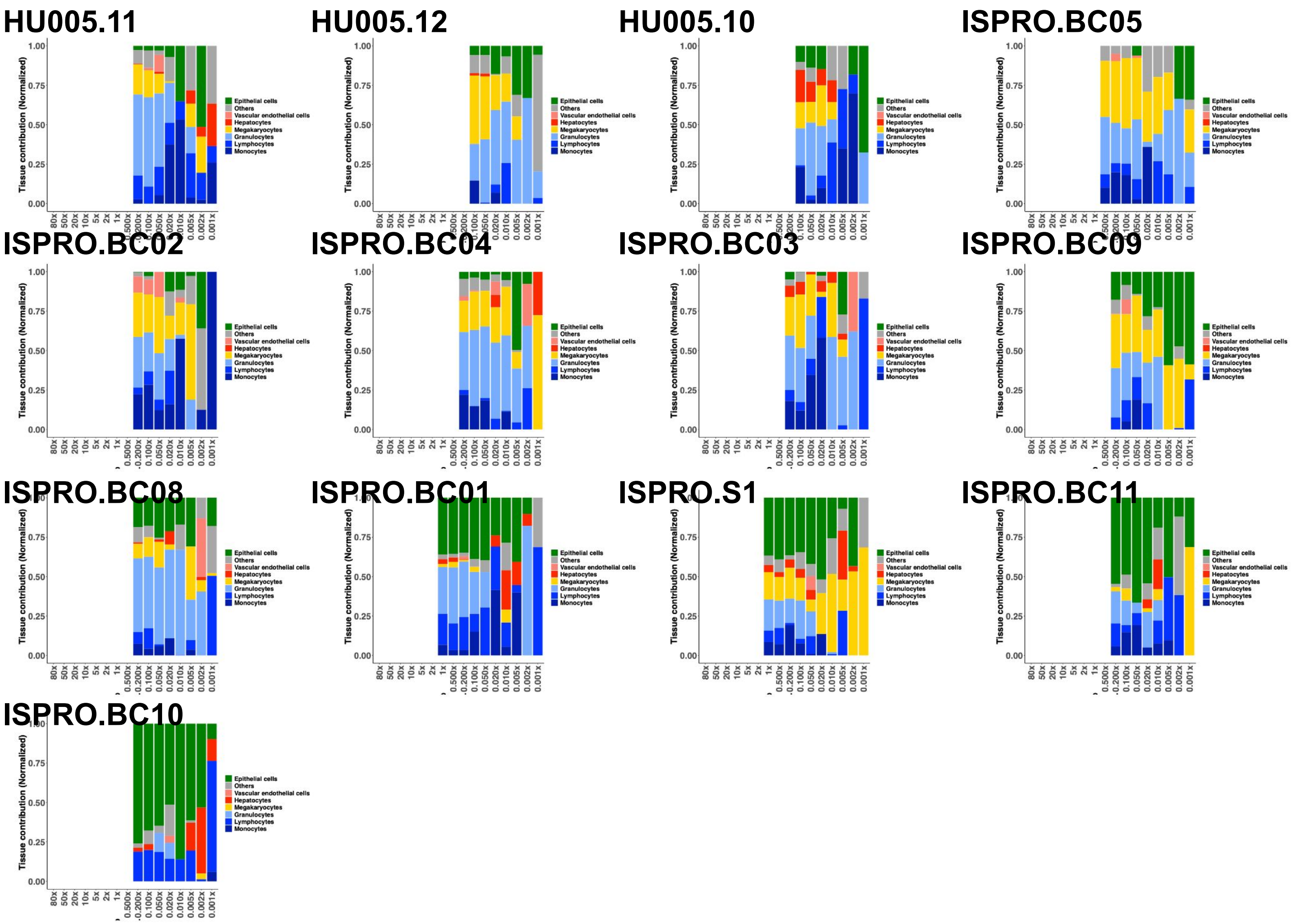

Figure S3: DNA methylation deconvolution for cfNano samples. Each cfNano sample from the current study was downsampled from full depth to 0.001x coverage, and sample ordering is the same as Fig 1B-C. Short names are used, and full sample information is available in Supplementary Table 1.

Fig S4

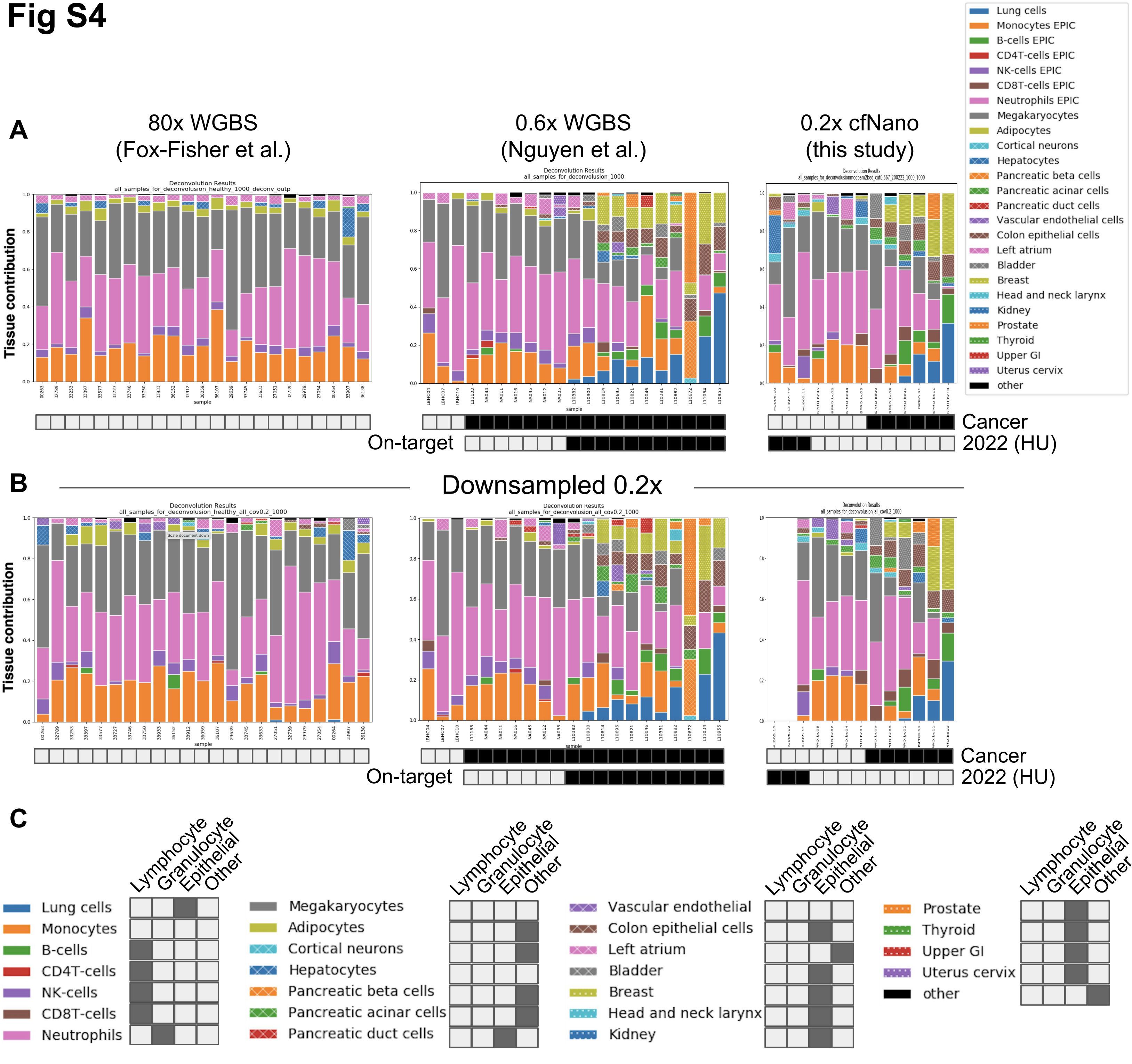

Figure S4: Full cell type assignments in deconvolution analysis. (A) Cell-type deconvolution for WGBS and cfNano datasets, using 25 cell types from [3]. (B) 25 cell type deconvolution of all samples downsampled to 0.2x sequence coverage. (C) The four cell-type groups from Figure 1 (Lymphocyte, Granulocyte, Epithelial, and Other) and which of the 25 cell types were collapsed into each group. All cell types not assigned to one of the four groups are shown as a singleton cell type in Figure 1.

Fig S5

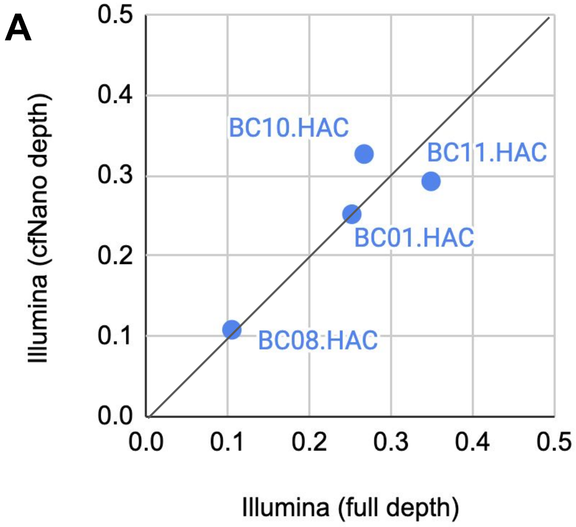

Figure S5: ichorCNA tumor fractions of downsampled Illumina samples. Four Illumina plasma samples from LuAd patients are shown. ichorCNA tumor fraction was computed at full sequence depth (x axis) and by randomly downsampling the Illumina samples to have the same number of fragments as the corresponding cfNano sample.

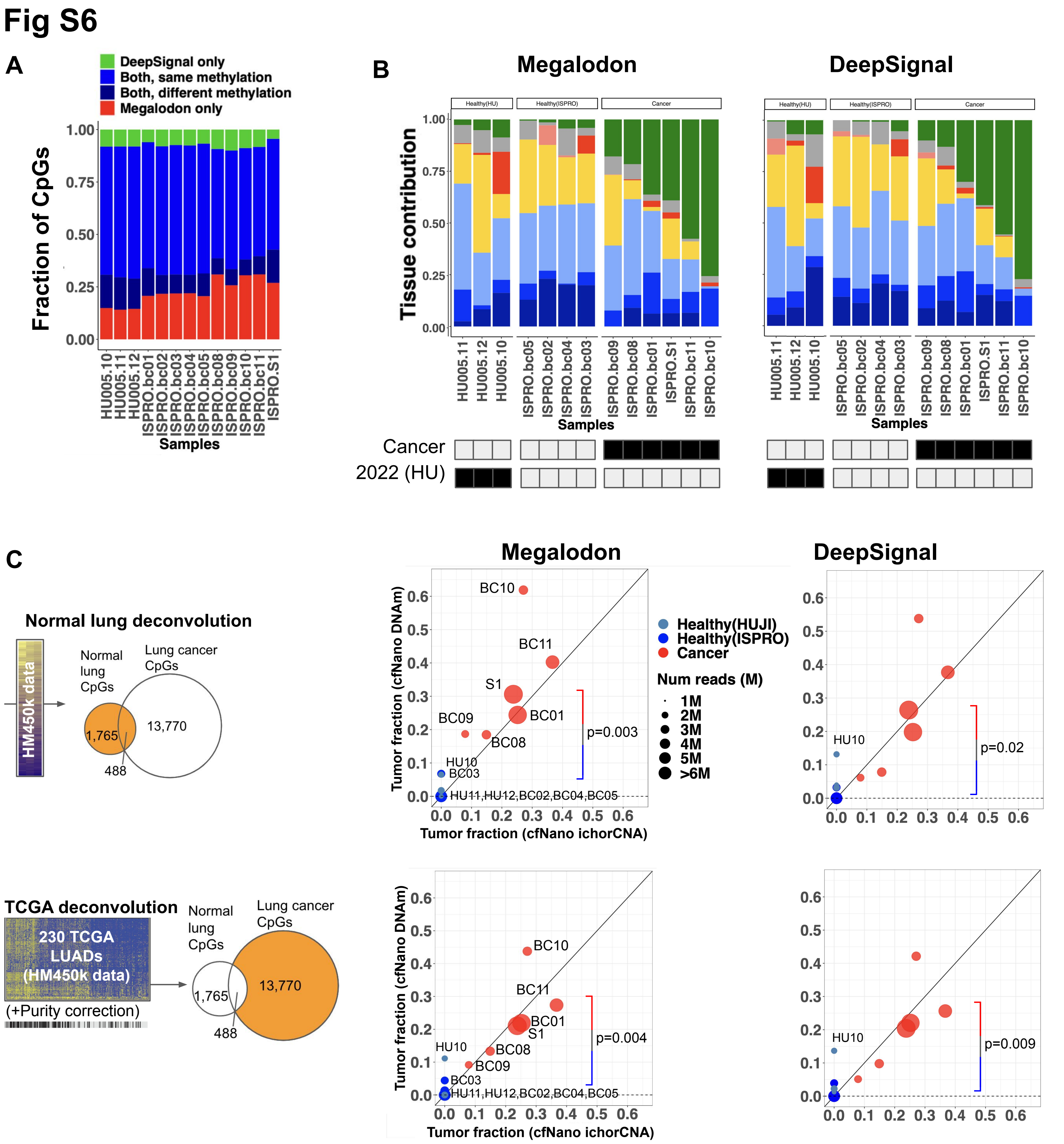

Figure S6: Calling cfNano methylation with two different methods. (A) DeepSignal and Megalodon were used to call CpG methylation for each cfNano sample. CpGs were divided into those covered by DeepSignal only, Megalodon only, or Both. Those covered by both were divided into those that got identical methylation status vs. different methylation status. (B) Grouped cell type deconvolution is shown for all samples for Megalodon and DeepSignal processed data. Megalodon version is reproduced from Figure 1B. (C) Two-component deconvolution is shown for all samples for Megalodon and DeepSignal processed data. Megalodon versions are reproduced from Figure 1F and 1G, respectively.

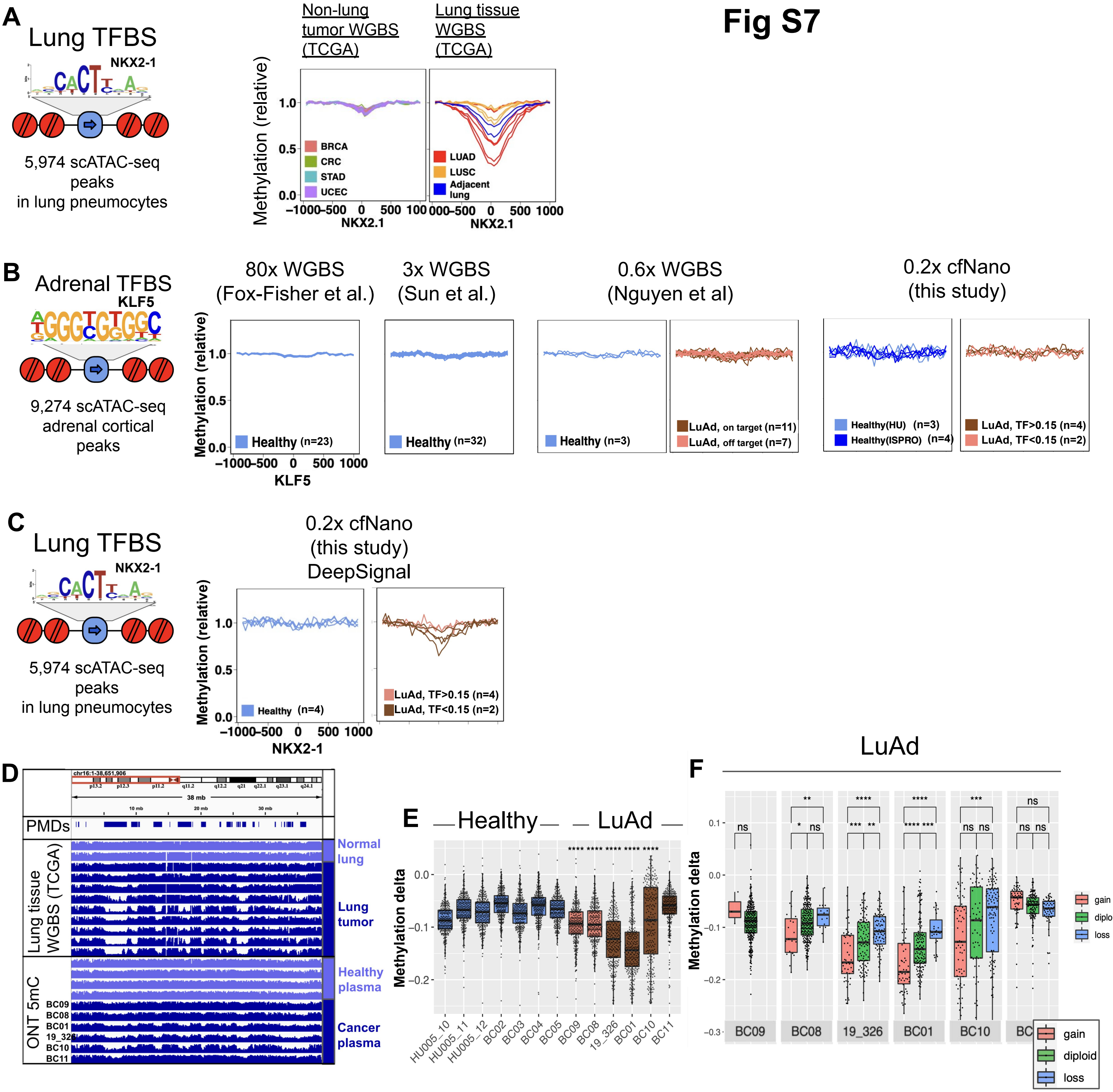

Figure S7: Genomic context of DNA methylation changes. (A) Methylation in 18 TCGA WGBS non-lung tumors (left) and 11 TCGA WGBS lung tumors and adjacent normal tissue (right) from [4]. Plasma cfDNA methylation levels were averaged from -1kb to +1kb relative to 5,974 pneumocyte-specific NKX2-1 transcription factor binding sites (TFBS) taken from [5]. All methylation values are shown as relative to the flanking region (from 0.8kb-1kb relative to TFBS). (B) 9,274 adrenal cortical cell specific KLF5 TFBS taken from [5]. From left to right, plots show 23 healthy plasma samples from [1] and 32 healthy plasma samples from [6], followed by 3 healthy and 18 LuAd WGBS samples from [2] and 7 healthy and 6 LuAd cfNano samples from this study (C) cfNano methylation levels for lung NKX2-1 (same as Figure 2A), using DeepSignal methylation calling. (D) IGV analysis (same as Figure 2B) using DeepSignal methylation calling. (E-F) Genome-wide PMD bin analysis (same as Figure 2C-D) using DeepSignal methylation calling.

Figure S8

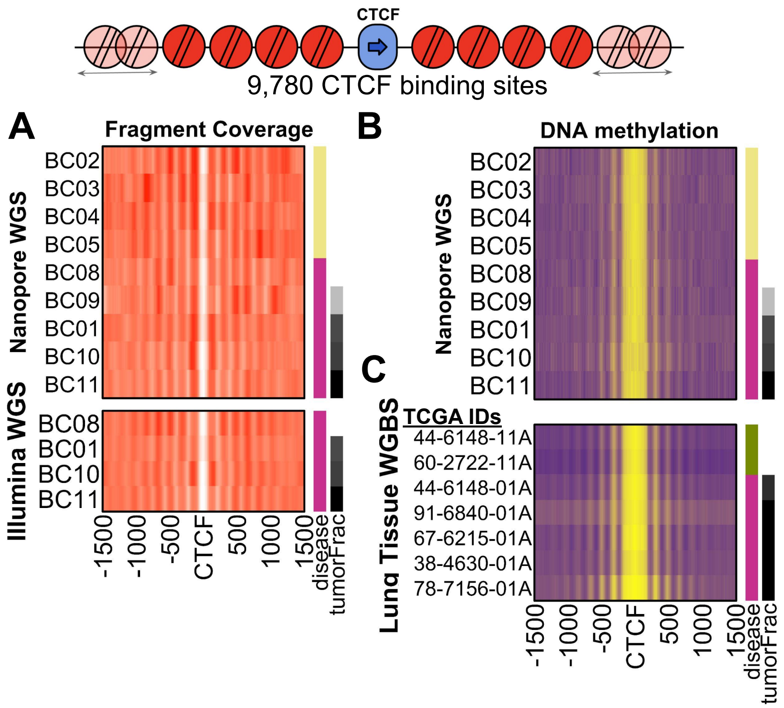

Figure S8: cfNano preserves fragmentomic and DNA methylation markers of nucleosome positioning. Alignments to CTCF motifs within 9,780 distal ChIP-seq peaks from [7]. (A, top) cfDNA fragment coverage shown as fold-change vs. average coverage depth across the genome. The plot includes only fragments of length 130-155bp to maximize resolution. (A, bottom) Matched Illumina samples of higher sequencing depth (median 17.0M fragments in Illumina vs. 6.4M in ONT samples). (B) CTCF DNA methylation of Nanopore samples from this study at CTCF sites. (C) DNA methylation from seven lung tissue WGBS samples from TCGA [4].

Figure S9

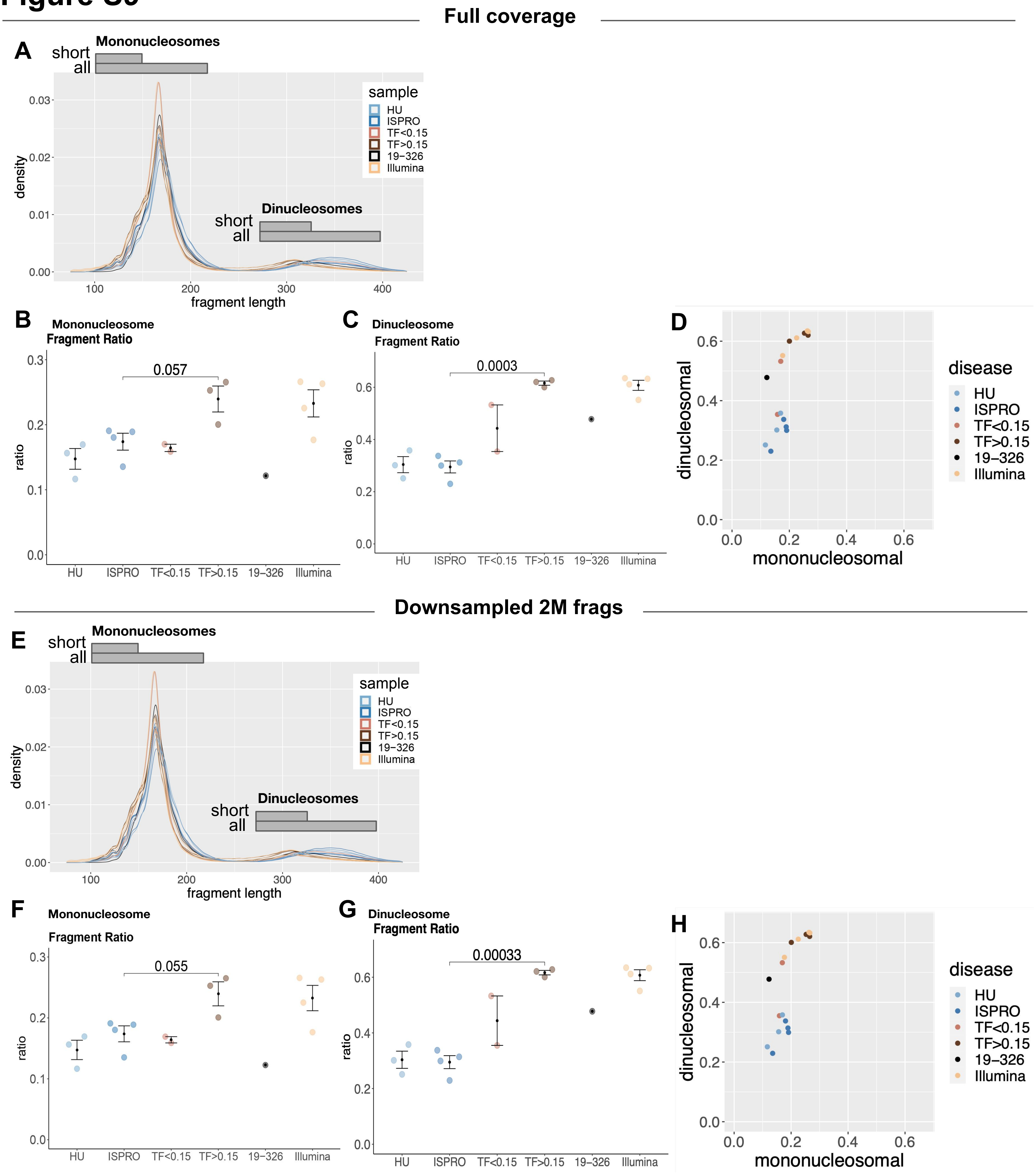

Figure S9: Effects of downsampling on fragment length of cfNano and Illumina WGS. (A-C) Data from Figure 4A,B,D are reproduced with the addition of sample 19_326 (which used a different, non-barcoded, cfNano adapter design), as well as matched Illumina samples. (D) Short mononucleosome ratios (x axis) plotted against short dinucleotide ratios (y axis). Panels (E-H) show the same plots as panels A-D, but with each sample randomly downsampled to 2M fragments. Statistical significance levels for panels B,C,F, and G were determined by two-tailed t-test.

Figure S10

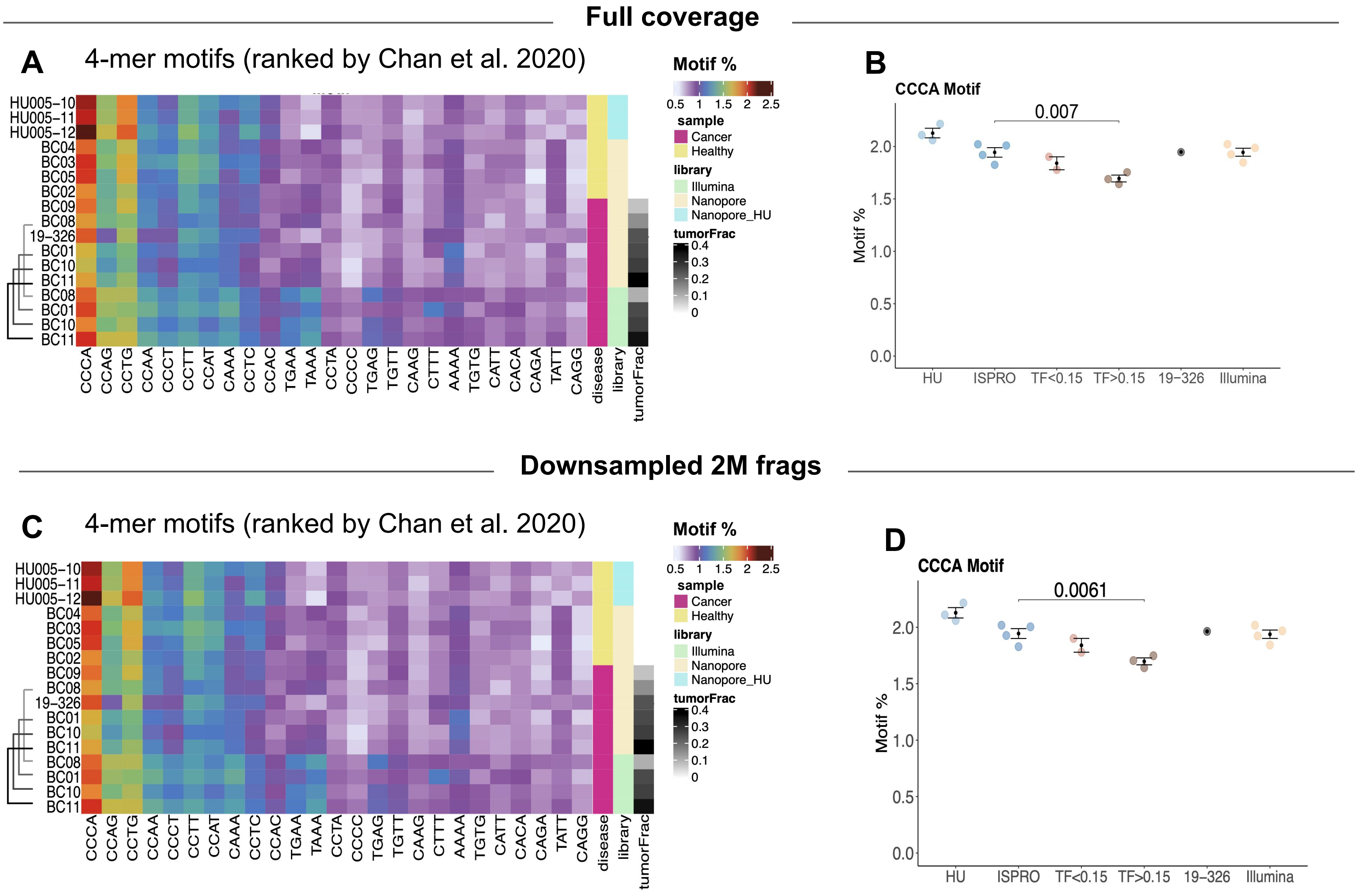

Figure S10: Effects of downsampling on fragment end features of cfNano and Illumina WGS. (A-B) are reproduced from main Figure 4F and 4I, with the addition of sample 19_326 (which used a different, non-barcoded, cfNano adapter design), as well as matched Illumina samples. Panels (C-D) show the same plots, but with each sample randomly downsampled to 2M fragments.Statistical significance levels for panels B and D were determined by two-tailed t-test.
