# Supplementary material for: Detecting cell-of-origin and cancer-specific methylation features of cell-free DNA from Nanopore sequencing
Source: Genome Biol. 2022 Jul 15;23:158. doi: 10.1186/s13059-022-02710-1 (PMC9283844; doi:10.1186/s13059-022-02710-1)
Supplement: Supplementary file 5 — Additional file 5. ichorCNA plots for all cfNano and matched Illumina WGS samples. [file 13059_2022_2710_MOESM5_ESM.zip › ichorCNA-cfNano-original.pdf]

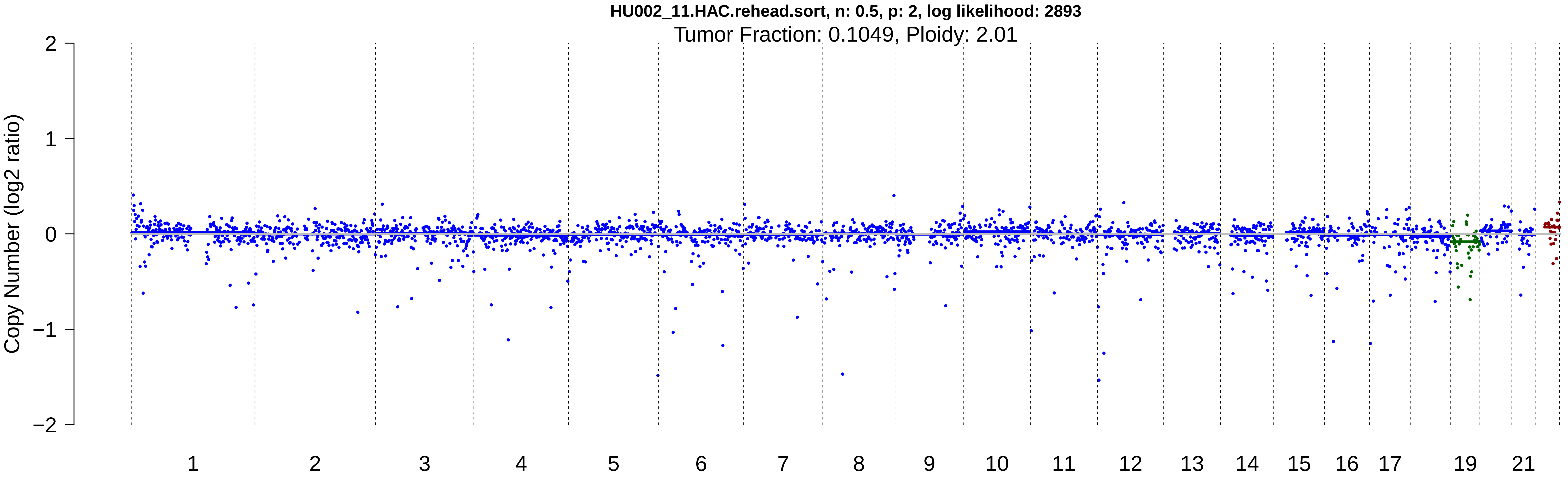
HU002_11.HAC.rehead.sort, n: 0.5, p: 2, log likelihood: 2893
Tumor Fraction: 0.1049, Ploidy: 2.01
Copy Number (log2 ratio)
2
1
0
−1
−2
1
2
3
4
5
6
7
8
9
10
11
12
13
14
15
16
17
19
21

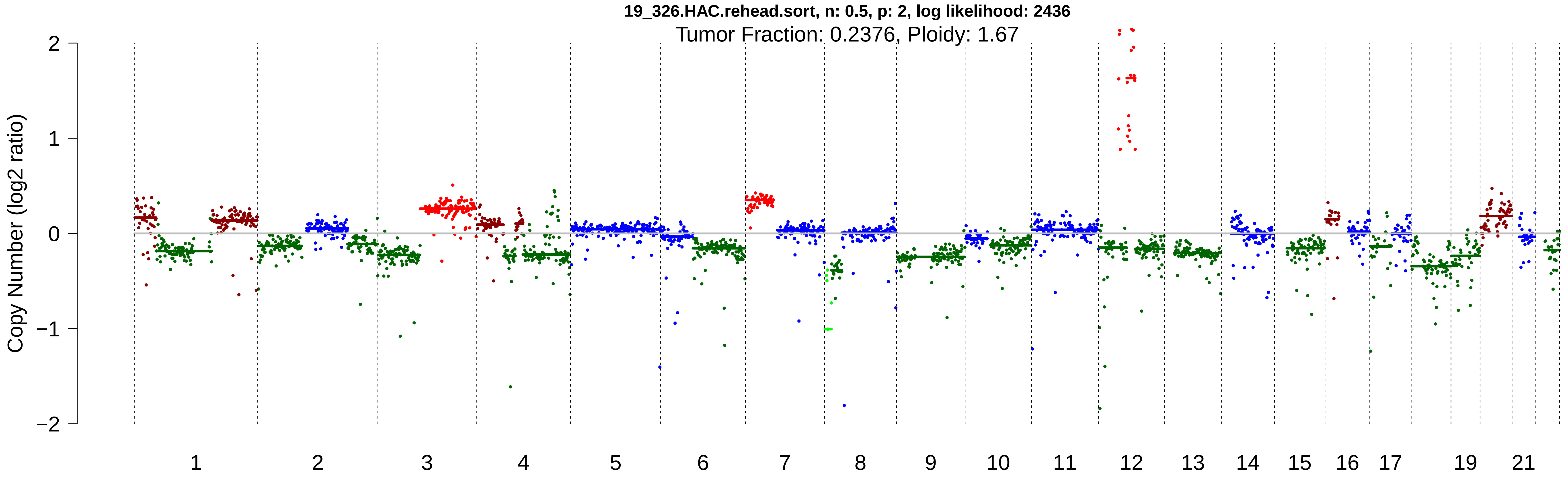

19_326.HAC.rehead.sort, n: 0.5, p: 2, log likelihood: 2436
Tumor Fraction: 0.2376, Ploidy: 1.67
Copy Number (log2 ratio)
2
1
0
−1
−2
1
2
3
4
5
6
7
8
9
10
11
12
13
14
15
16
17
19
21

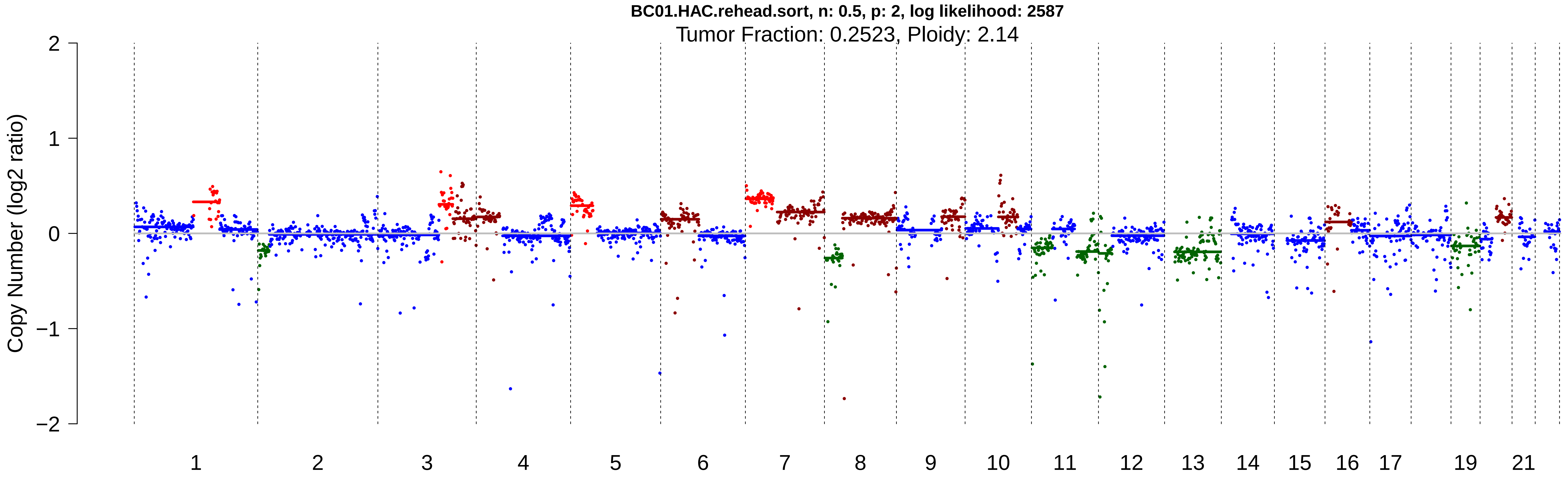
BC01.HAC.rehead.sort, n: 0.5, p: 2, log likelihood: 2587
Tumor Fraction: 0.2523, Ploidy: 2.14
Copy Number (log2 ratio)
2
1
0
−1
−2
1
2
3
4
5
6
7
8
9
10
11
12
13
14
15
16
17
19
21

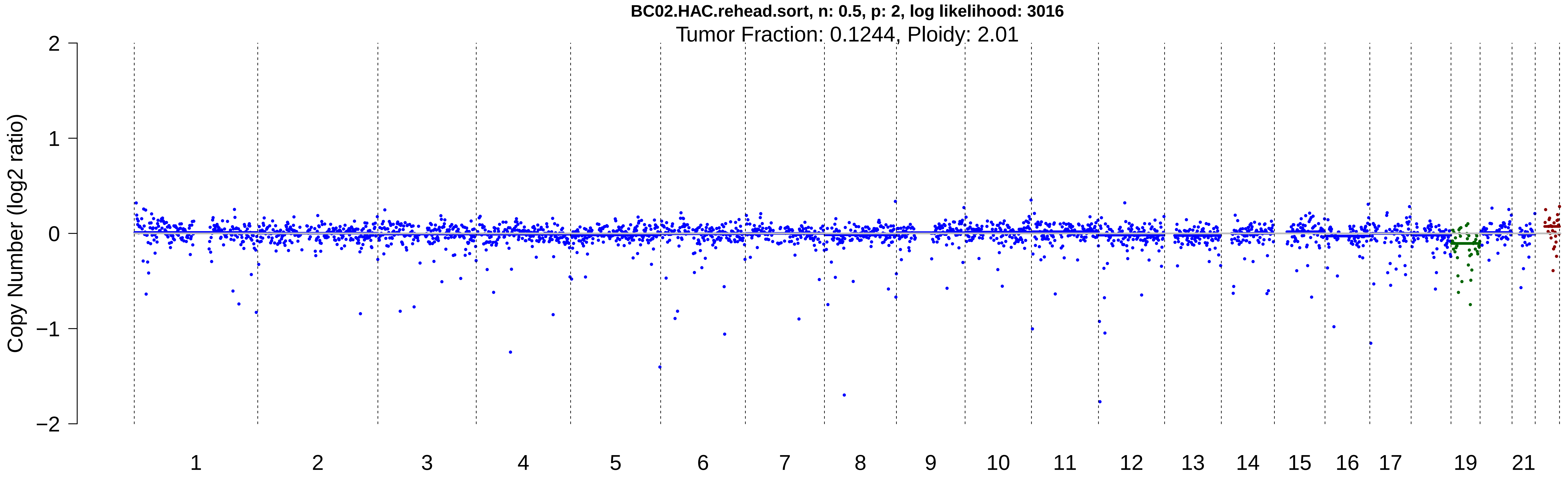
BC02.HAC.rehead.sort, n: 0.5, p: 2, log likelihood: 3016
Tumor Fraction: 0.1244, Ploidy: 2.01
Copy Number (log2 ratio)
2
1
0
−1
−2
1
2
3
4
5
6
7
8
9
10
11
12
13
14
15
16
17
19
21

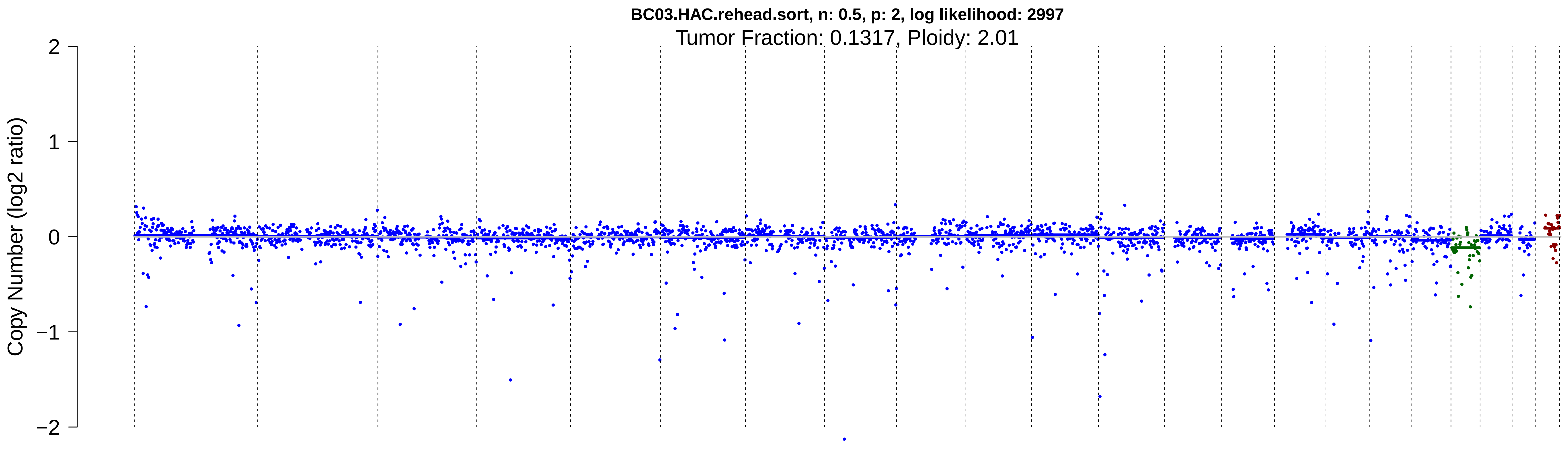
BC03.HAC.rehead.sort, n: 0.5, p: 2, log likelihood: 2997
Tumor Fraction: 0.1317, Ploidy: 2.01
Copy Number (log2 ratio)
2
1
0
−1
−2
1
2
3
4
5
6
7
8
9
10
11
12
13
14
15
16
17
19
21

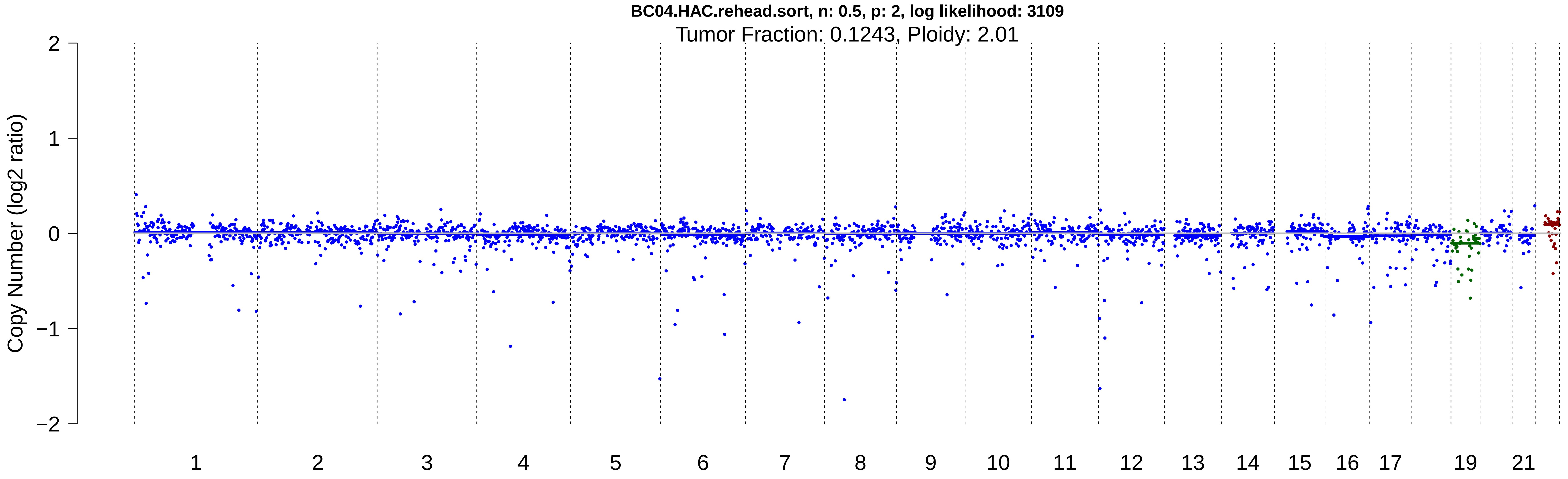

BC04.HAC.rehead.sort, n: 0.5, p: 2, log likelihood: 3109
Tumor Fraction: 0.1243, Ploidy: 2.01
Copy Number (log2 ratio)
2
1
0
−1
−2
1
2
3
4
5
6
7
8
9
10
11
12
13
14
15
16
17
19
21

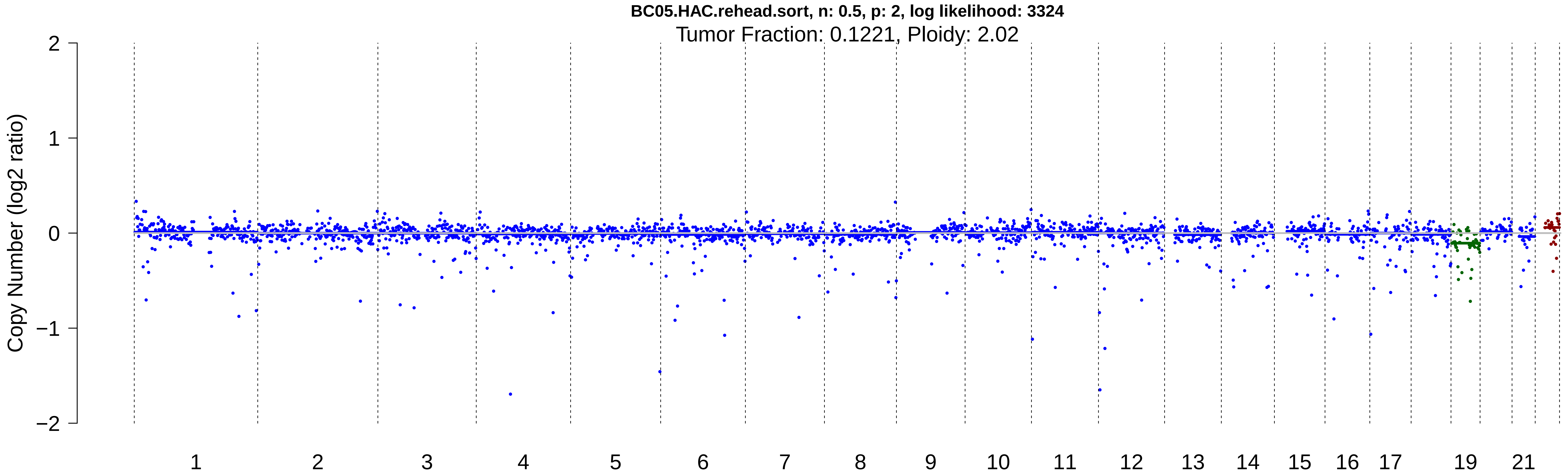

BC05.HAC.rehead.sort, n: 0.5, p: 2, log likelihood: 3324
Tumor Fraction: 0.1221, Ploidy: 2.02
Copy Number (log2 ratio)
2
1
0
−1
−2
1
2
3
4
5
6
7
8
9
10
11
12
13
14
15
16
17
19
21

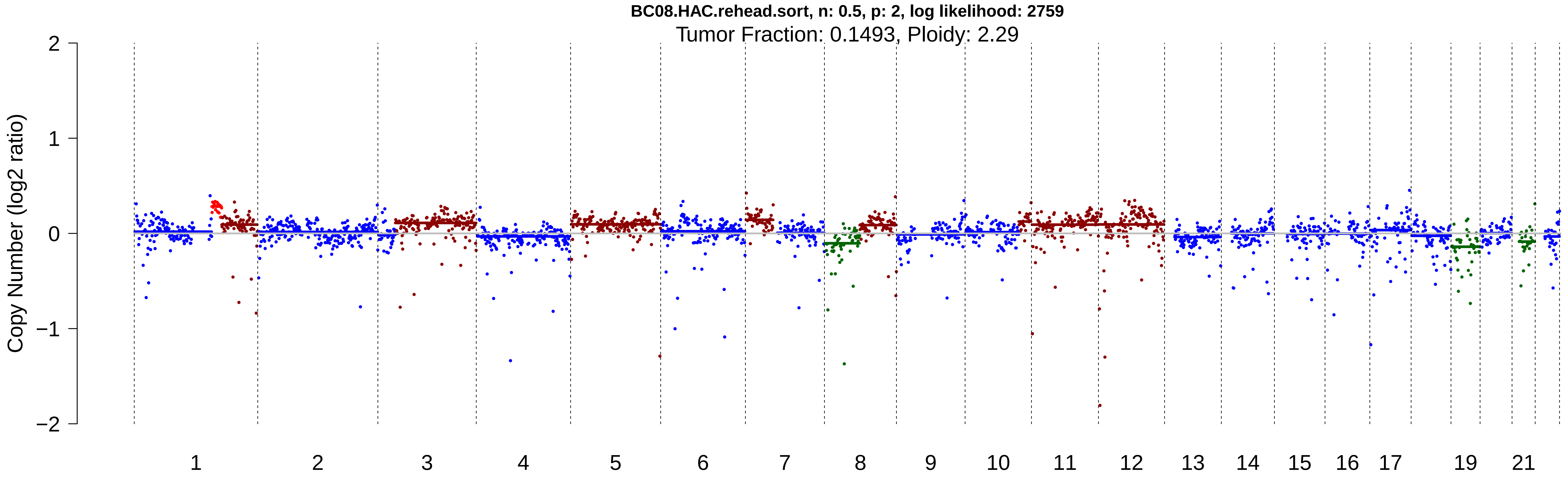

BC08.HAC.rehead.sort, n: 0.5, p: 2, log likelihood: 2759
Tumor Fraction: 0.1493, Ploidy: 2.29
Copy Number (log2 ratio)
2
1
0
−1
−2
1
2
3
4
5
6
7
8
9
10
11
12
13
14
15
16
17
19
21

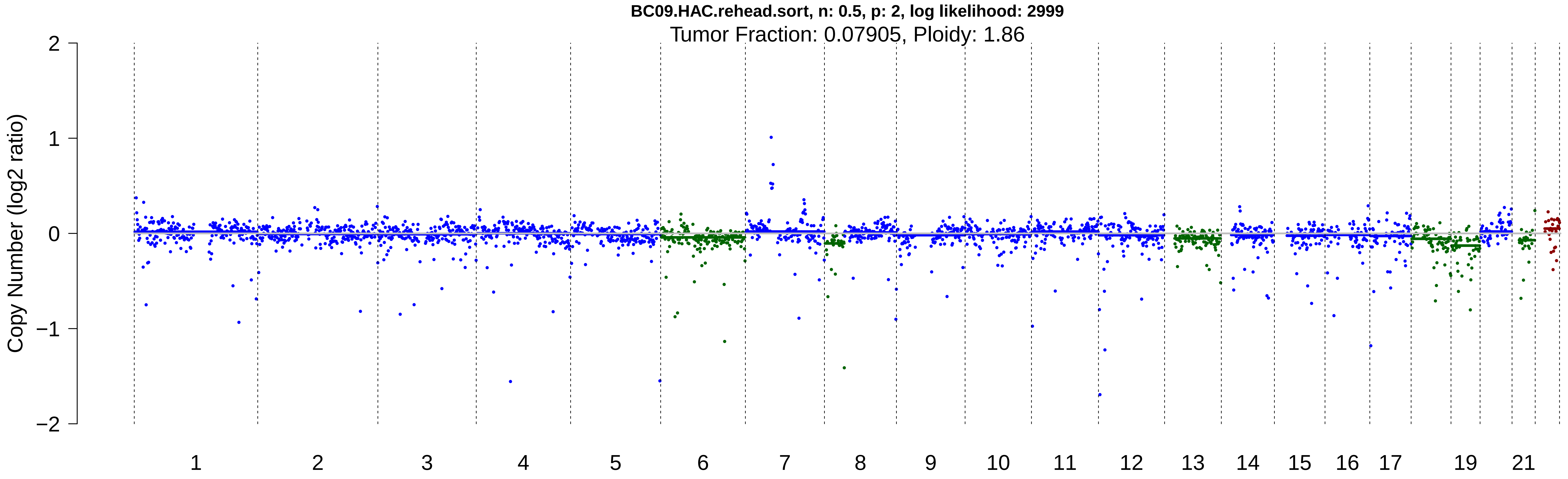
BC09.HAC.rehead.sort, n: 0.5, p: 2, log likelihood: 2999
Tumor Fraction: 0.07905, Ploidy: 1.86
Copy Number (log2 ratio)
2
1
0
−1
−2
1
2
3
4
5
6
7
8
9
10
11
12
13
14
15
16
17
19
21

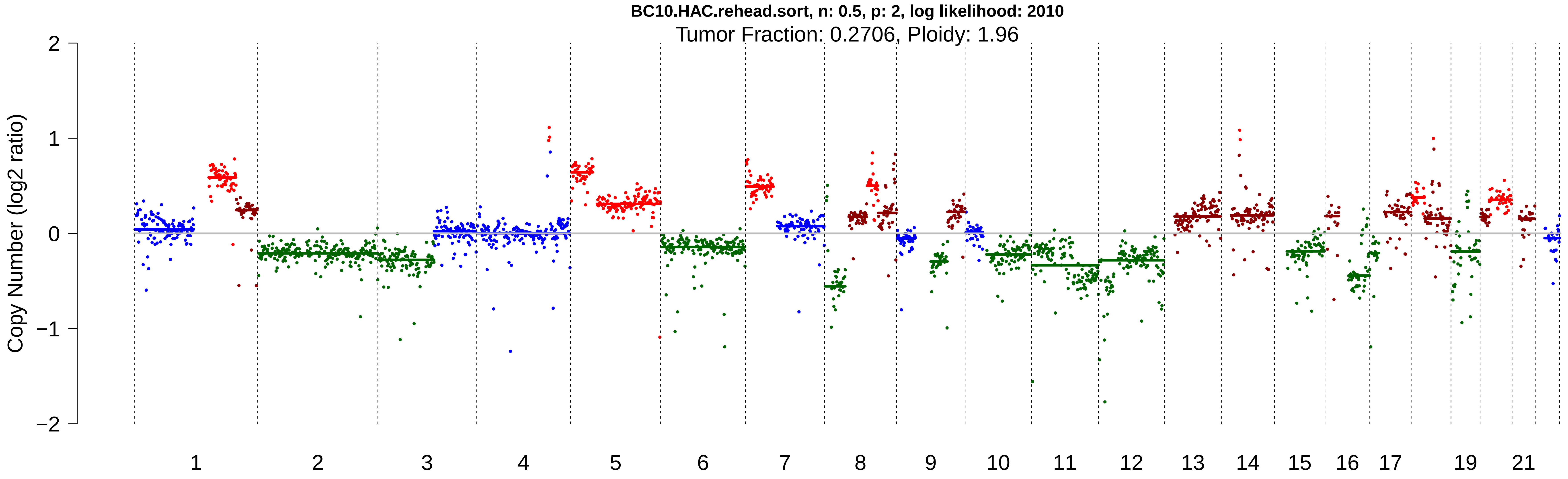
BC10.HAC.rehead.sort, n: 0.5, p: 2, log likelihood: 2010
Tumor Fraction: 0.2706, Ploidy: 1.96
Copy Number (log2 ratio)
2
1
0
−1
−2
1
2
3
4
5
6
7
8
9
10
11
12
13
14
15
16
17
19
21

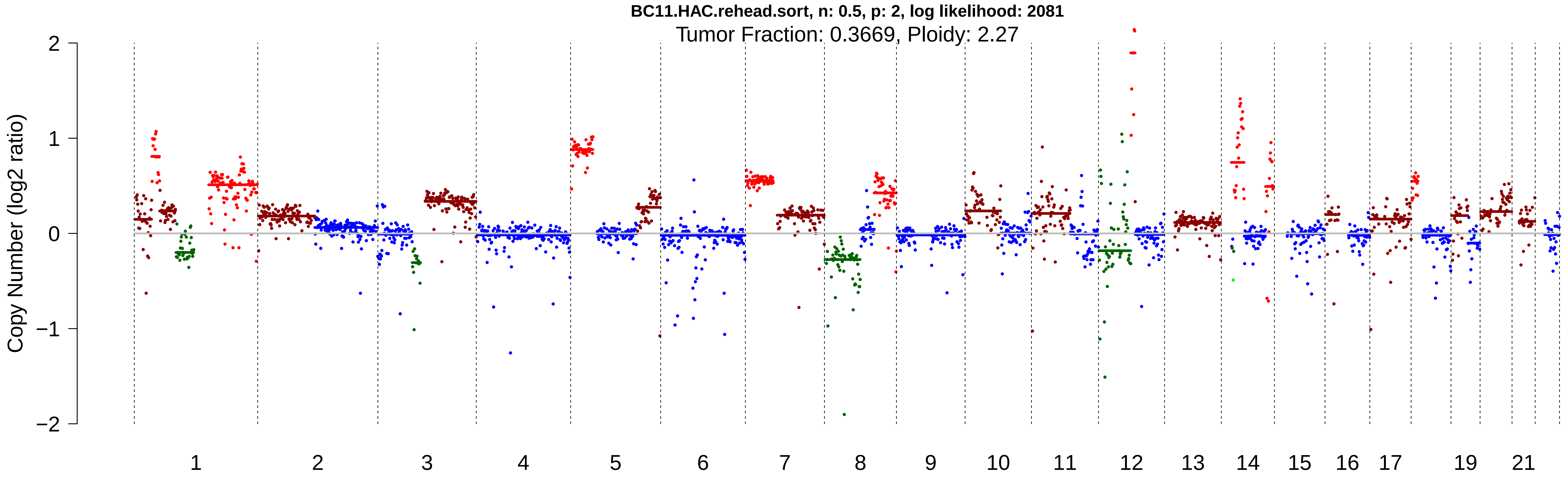
BC11.HAC.rehead.sort, n: 0.5, p: 2, log likelihood: 2081
Tumor Fraction: 0.3669, Ploidy: 2.27
Copy Number (log2 ratio)
2
1
0
−1
−2
1
2
3
4
5
6
7
8
9
10
11
12
13
14
15
16
17
19
21

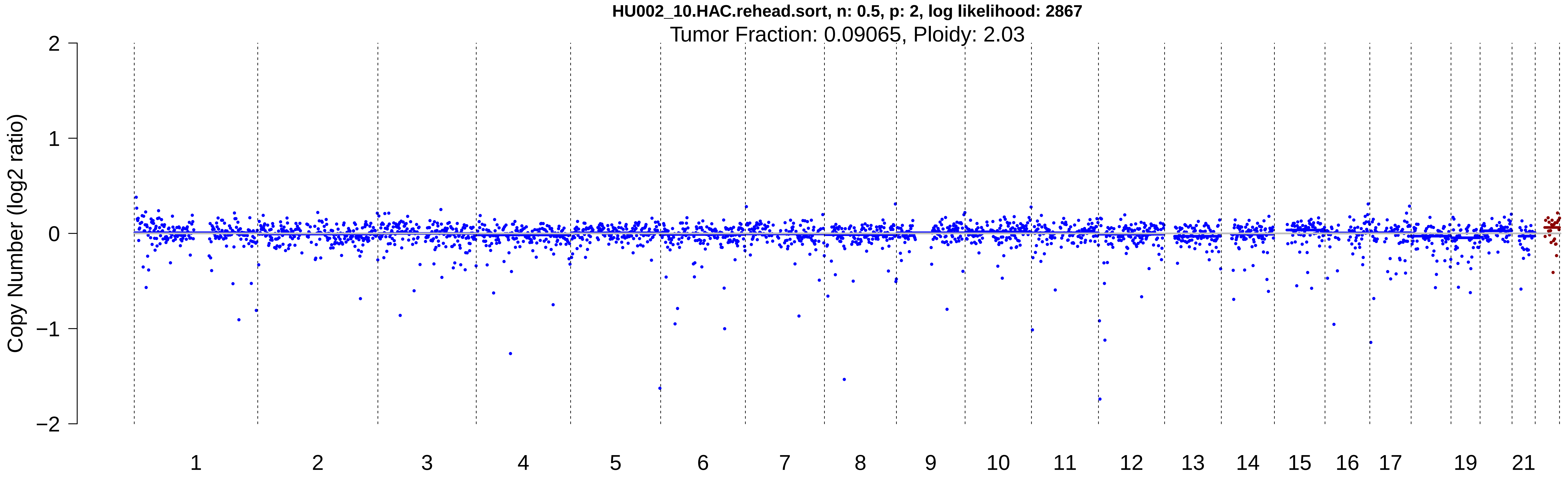
HU002_10.HAC.rehead.sort, n: 0.5, p: 2, log likelihood: 2867
Tumor Fraction: 0.09065, Ploidy: 2.03
Copy Number (log2 ratio)
2
1
0
−1
−2
1
2
3
4
5
6
7
8
9
10
11
12
13
14
15
16
17
19
21

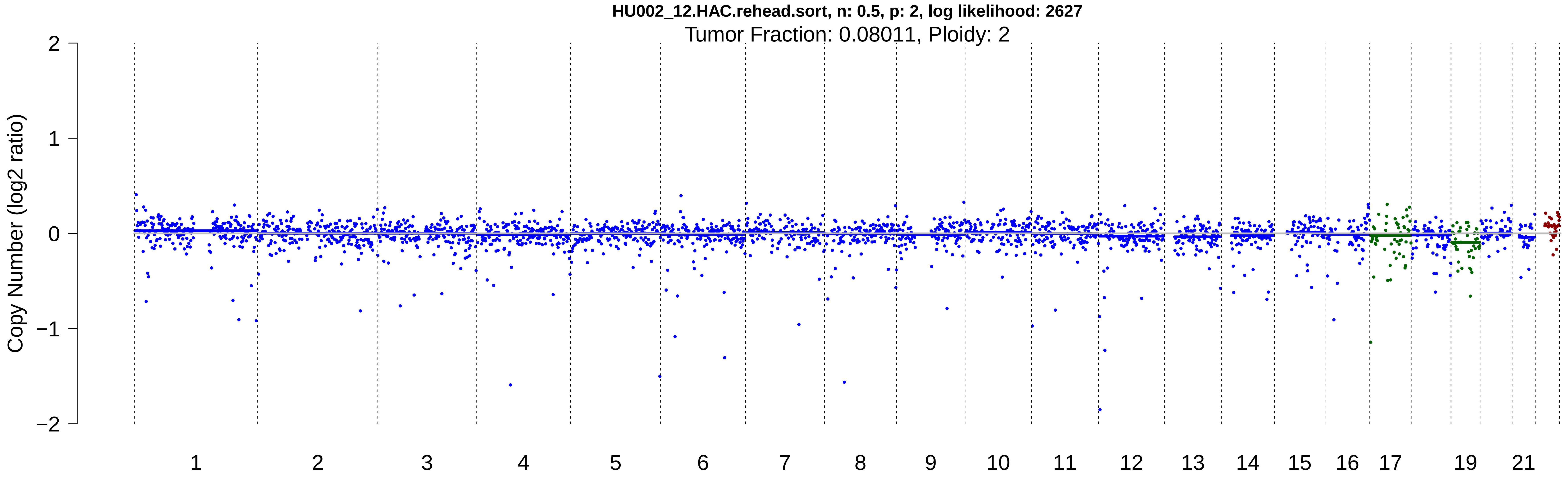
HU002_12.HAC.rehead.sort, n: 0.5, p: 2, log likelihood: 2627
Tumor Fraction: 0.08011, Ploidy: 2
Copy Number (log2 ratio)
2
1
0
−1
−2
1
2
3
4
5
6
7
8
9
10
11
12
13
14
15
16
17
19
21
