# Supplementary material for: Detecting cell-of-origin and cancer-specific methylation features of cell-free DNA from Nanopore sequencing
Source: Genome Biol. 2022 Jul 15;23:158. doi: 10.1186/s13059-022-02710-1 (PMC9283844; doi:10.1186/s13059-022-02710-1)
Supplement: Supplementary file 5 — Additional file 5. ichorCNA plots for all cfNano and matched Illumina WGS samples. [file 13059_2022_2710_MOESM5_ESM.zip › ichorCNA-Illumina-original.pdf]

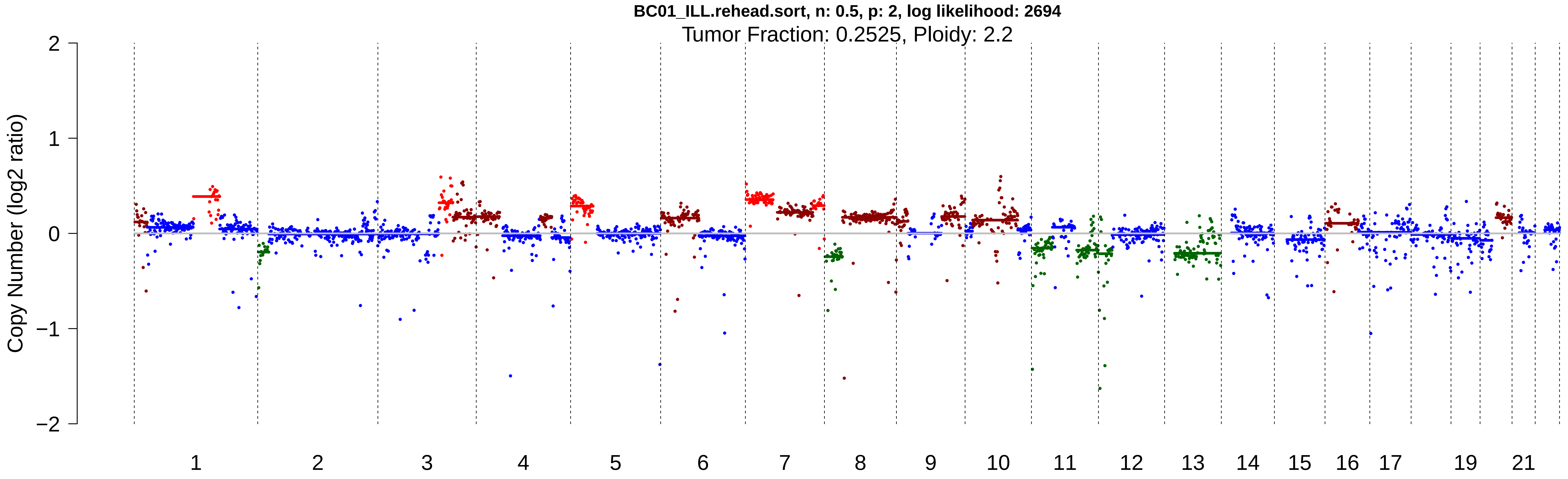
BC01_ILL.rehead.sort, n: 0.5, p: 2, log likelihood: 2694
Tumor Fraction: 0.2525, Ploidy: 2.2
Copy Number (log2 ratio)
2
1
0
−1
−2
1
2
3
4
5
6
7
8
9
10
11
12
13
14
15
16
17
19
21

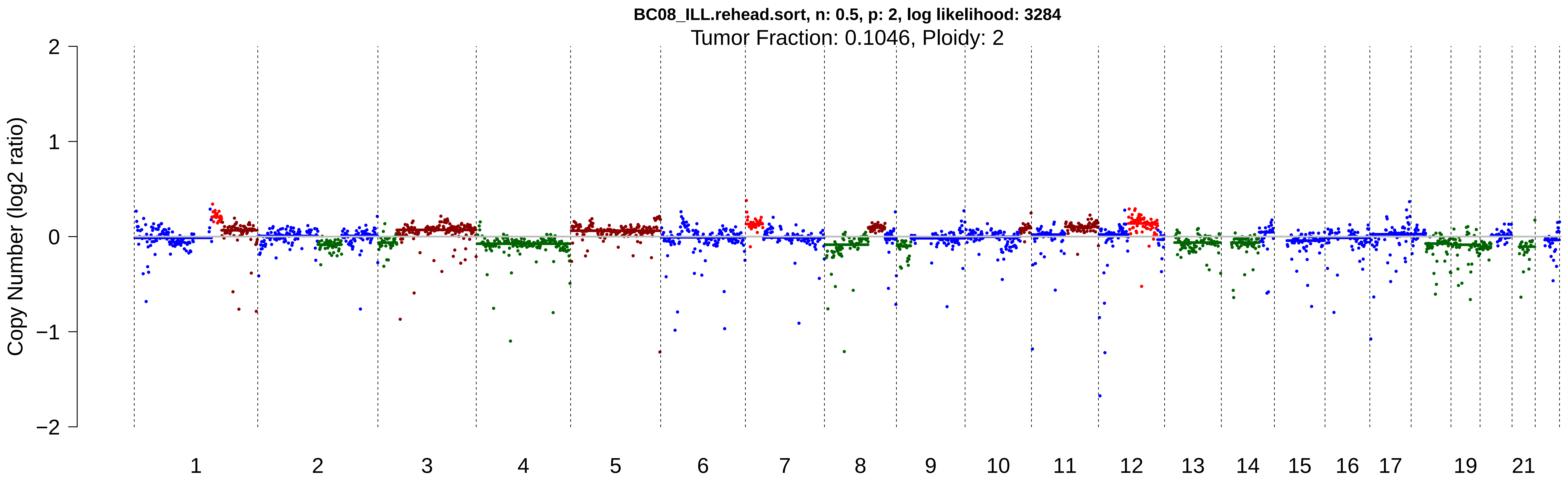
BC08_ILL.rehead.sort, n: 0.5, p: 2, log likelihood: 3284
Tumor Fraction: 0.1046, Ploidy: 2
Copy Number (log2 ratio)
2
1
0
−1
−2
1
2
3
4
5
6
7
8
9
10
11
12
13
14
15
16
17
19
21

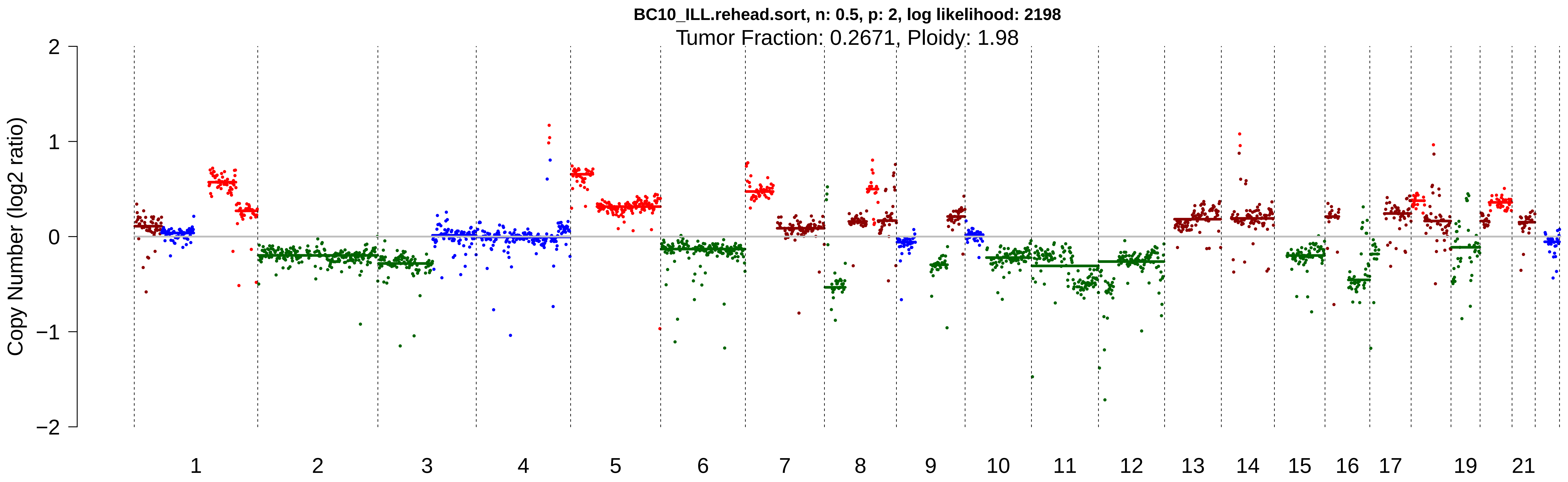

BC10_ILL.rehead.sort, n: 0.5, p: 2, log likelihood: 2198
Tumor Fraction: 0.2671, Ploidy: 1.98
Copy Number (log2 ratio)
2
1
0
−1
−2
1
2
3
4
5
6
7
8
9
10
11
12
13
14
15
16
17
19
21

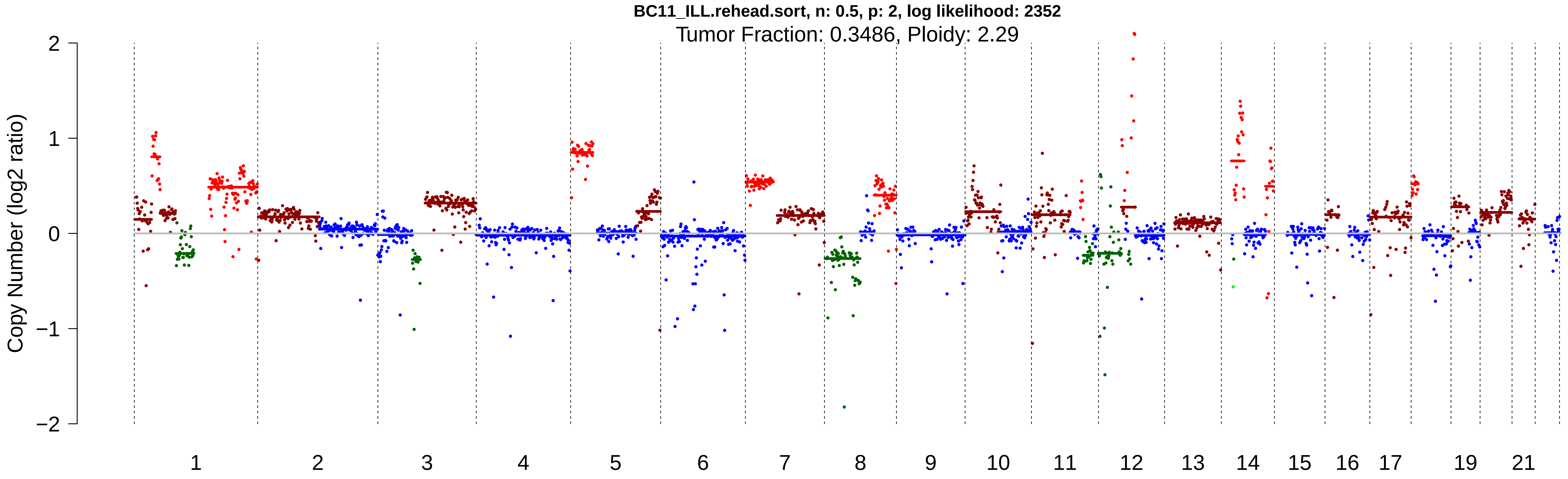
BC11_ILL.rehead.sort, n: 0.5, p: 2, log likelihood: 2352
Tumor Fraction: 0.3486, Ploidy: 2.29
Copy Number (log2 ratio)
2
1
0
−1
−2
1
2
3
4
5
6
7
8
9
10
11
12
13
14
15
16
17
19
21
