# Supplementary material for: Detecting cell-of-origin and cancer-specific methylation features of cell-free DNA from Nanopore sequencing
Source: Genome Biol. 2022 Jul 15;23:158. doi: 10.1186/s13059-022-02710-1 (PMC9283844; doi:10.1186/s13059-022-02710-1)
Supplement: Supplementary file 6 — Additional file 6. Review history. [file 13059_2022_2710_MOESM6_ESM.docx]

Review History

**First round of review**

**Reviewer 1**

**Are you able to assess all statistics in the manuscript, including the appropriateness of statistical tests used?** Yes, and I have assessed the statistics in my report.

**Comments to author:**

In this article, "Detecting cell-of-origin and cancer-specific features of cell-free DNA with nanopore sequencing," Katsman, Orlanski, and Martignano et al. demonstrated some proof-of-concept whole genome sequencing from circulating cell-free DNA using conventional Oxford Nanopore Technologies (ONT) sequencing, showing it can detect cell-of-origin and cancer-specific 5mC changes reasonably well, while also revealing "fragmentomic" data. They propose this could be useful for ctDNA diagnostics as well as non-cancer applications in emergency medicine. The authors are correct that the ONT platform has finally come to a reasonable maturity and can now be used for these purposes, and overall this study is a useful addition to the field, but some questions remain:

1) Can the authors show this utility on more data, even if public data, to show negative signals? The paper uses a small data set, sampling 4mL of plasma for four healthy control individuals and six metastatic lung adenocarcinoma cases, but this is a small cohort.
2) Do they see any difference in the accuracy or utility of the mC sites if they use a different base caller, like Megalodon? Making the claims on these sites is likely somewhat dependent on the base caller they used (DeepSignal), and so this should be teased out, validated, and quantified.
3) The authors noted that "ATAC-seq open chromatin regions are almost universally demethylated in cancer," and they also saw this in their samples, but did they observe the opposite trend in closed sites across the genome as well?
4) They found that the lung specificity of the NKX2-1 sites was quite distinct, which makes sense given it is a transcription factor in lung pneumocytes. Are there other lung-specific signatures they could use to validate this trend?
5) Related to #4, do the authors find any novel sites that might be differentiating between the lung tissue and normal? Knowing they are underpowered, this would just be exploratory, but it would show the potential of the method to discover new markers and regulatory regions.
6) The default software for ONT does not allow fragments below 200nt; did the authors need to adjust the run-time software to account for this, or make any other modifications to the ONT flowcell processing?
7) The authors mention that "higher read depth will be necessary to detect nucleosomal fragmentation patterns from cell-type specific components of cfDNA," but some down-sampling or estimate of this would be good to see, and in general, to help guide future experiments in this direction. Their conclusions recommend a mean of 6.4M reads, but it would be nice to know how low we can go (2.6M seems a little low, for example in BC10), and if 3-4M reads might be enough. A deconvolution accuracy with incrementing sets of 1M reads would be very illustrative and is recommended.
8) There is one additional benchmarking study they should reference that supports their claims and also observed that the motif differences they observe between Illumina and ONT could be a function of the ONT base caller: Foox et al., Genome Biology, 2021. Also, a table of their other predicted motifs would be useful.

**Reviewer 2**

**Are you able to assess all statistics in the manuscript, including the appropriateness of statistical tests used?** No, I do not feel adequately qualified to assess the statistics.

**Comments to author:**

In the article entitled "Detecting cell-of-origin and cancer-specific features of cell-free DNA with Nanopore sequencing" Katsman et al describe an approach based on Nanopore sequencing for analyzing epigenetic features of cell-free DNA (cfDNA) in plasma. In particular, using nanopore sequencing could allow to by-pass the degradation linked to bisulfite conversion to analysis 5mC in minute amounts of cfDNA.

The authors major claims, that Nanopore sequencing can retrieve cell of origin and cancer specific features on cell-free DNA, are only partially supported by the results presented.
1. The sensitivity and accuracy for determining the cell of origin of cfDNA is not concretely determined. The authors are not using any form of positive control to determine the accuracy for determining the cell of origin of cfDNA in plasma (e.g. what fraction of white blood cell is detected using Nanopore, how does this compare to a control, is it better or worse than Illumina based approaches?). Viewing this is a feasibility study, I am not convinced comparison with database will be enough to accurately determine such populations.
2. The size of the cohort of matched Illumina and ONT samples is small. Even if this is a feasibility study, the manuscript results would be more convincing with a larger cohort of matched Illumina-Nanopore cases.
3. The results in figure 2 are not supporting the claim that Nanopore is preserving cfDNA fragmentation features. Even if Illumina and Nanopore data are vaguely compared, a much more throughout point-by-point quantitative comparison per matched samples on various fragmentomic features is needed to draw such strong conclusions. Is the signal maintained when coverage is reduced?
4. One figure has 14 panels, the other one 7 panels. I think the authors should do an effort to be as concise and specific as possible and move to supplementary materials and anecdotal or minimally processed results. This could increase the clarity and potential impact of the manuscript.
5. In general, the claimed added value of Nanopore sequencing versus Illumina seem anecdotal viewing the potential negative sides (or at least limitations, which should be discussed more in depth). In particular the sensitivity for detecting scarce signal might be lower than Illumina and potential for multiplexing (and thus cost/sample) higher. I agree that the potential of Nanopore for providing quickly results render the technology conceptually superior to Illumina for emergency medicine, but some direction of applications (or even better, preliminary data) could enhance the work.

I have also the following comments:
- Line 34: Conventional ONT sequencing is vague and misleading, as the reader most certainly understands amplicon-based sequencing. Please clarify that you used native DNA sequencing.
- Lines 48-50: The authors write about detecting unusual cell types in cfDNA, but this is out of context. Please add one line before this to highlight the expected cell type composition detectable from cfDNA.
- Line 78: Please mention the detection limit of ichorCNA explicitly in numbers.
- Lines 88-91: Please add the mean and SD methylation levels for controls and cancer samples.
- Lines 190-195: Please add the mean and SD methylation levels for controls and cancer samples.
- Lines 210-211. Please say specifically the direction of the difference for CCAA. Also mention the relatively high error rate of the ONT technology, and that you filtered soft-clipping.
- Materials and methods, lines 153-156: Please clarify if you extracted the genomic sequence or the actual read sequence for the analysis? If the latter, did you take any measures to filter reads with unmapped single, double or triple bases in the 4-mer? If the latter, please provide a supplementary figure showing the proportion of reads with soft-clipping, unmapped bases and fully mapping bases in their 5' 4-mers.
- Figure 2C: please use colorblind friendly colors in the heatmap.

**Authors Response**

**Point-by-point responses to the reviewers’ comments:**

Original editor/reviewer comments are in black, followed by author responses in blue. We cite line numbers in manuscript without page numbers, because lines are numbered consecutively throughout the manuscript document.

As you will see from the reports, both referees are broadly favorable and find the work of potential interest, but they raise important issues that we must ask you to address, in the form of a revised manuscript, before we reach a final decision on publication.

We are considering your manuscript as a 'Method' article. This is our format for publishing articles that describe a methodological innovation that is a significant advance over published methods and likely to be of broad utility, but that do not provide significant biological insights. When revising the manuscript, please ensure the manuscript conforms to our style for Methods articles (see https://genomebiology.biomedcentral.com/submission-guidelines/preparing-your-manuscript/method); specifically, the abstract should be under 100 words. {Please note that if we decide to publish your manuscript we will require that the source code is made publicly available under an open source license compliant with Open Source Initiative, with the license clearly stated in the manuscript. The source code should be deposited in a public repository, such as for instance github, with the accession links included in the manuscript. We also ask that the version of source code used in the manuscript is deposited in a DOI-assigning repository, such as zenodo, with the link also included. All this information should be listed in a separate Availability of Data and Materials section of the manuscript.}

We appreciate all editorial and reviewer comments, which have led to a substantially improved manuscript including new datasets and new analyses. We had every intention to complete this revision earlier, but almost each core author has been significantly affected in some way by the Omicron COVID waves.

Based on the reviewer comments, we added several important new components:

- A new methylation-based deconvolution analysis to show cell type breakdown of healthy samples and investigate effect of different sequence coverages
- Three new cfNano healthy samples sequenced at a second source site. These are now included in all analyses and are compared with the original cfNano samples (unfortunately we were not able to obtain new cancer samples)
- Incorporation of newly published Illumina WGBS cfDNA datasets, including very deep coverage of healthy individuals, and sequencing of lung adenocarcinoma cases (the same cancer type we sequenced with cfNano). These are included in all methylation analyses.
- Systematic fragmentomic comparisons of four LuAd samples that have matched cfNano and Illumina WGS libraries. These were not extensively compared in the first submission, and it provides an important benchmark. It also led to a new finding that dinucleosome fragment lengths may be more predictive of cancer than the commonly used mononucleosome fragment lengths.

Fortunately, the reviewer suggestions led to a much stronger paper. Unfortunately for the reviewers, we ended up rewriting large sections of the paper and reorganizing the figures to accommodate all the new content. We have highlighted sections of the text in blue that describe completely new analyses, but other sections have also been edited and improved. Almost every figure panel changed slightly because of the addition of three additional healthy samples, but we highlighted the completely new analyses in blue in the figure legends. We apologize to the reviewers and editors for the additional time it will take to review all the new text.

We have deposited all source code in GitHub repositories as described in the Code Availability section. We have also deposited static versions at Zenodo DOI 10.5281/zenodo.6445784.

Point by point responses follow below.

Reviewer #1

In this article, "Detecting cell-of-origin and cancer-specific features of cell-free DNA with nanopore sequencing," Katsman, Orlanski, and Martignano et al. demonstrated some proof-of-concept whole genome sequencing from circulating cell-free DNA using conventional Oxford Nanopore Technologies (ONT) sequencing, showing it can detect cell-of-origin and cancer-specific 5mC changes reasonably well, while also revealing "fragmentomic" data. They propose this could be useful for ctDNA diagnostics as well as non-cancer applications in emergency medicine. The authors are correct that the ONT platform has finally come to a reasonable maturity and can now be used for these purposes, and overall this study is a useful addition to the field, but some questions remain:

1)	Can the authors show this utility on more data, even if public data, to show negative signals? The paper uses a small data set, sampling 4mL of plasma for four healthy control individuals and six metastatic lung adenocarcinoma cases, but this is a small cohort.

We agree that sample size is too small to be conclusive, but we believe the new results are overwhelmingly positive and give enough confidence to justify larger studies. We added substantial new WGBS data to our DNA methylation analyses, most importantly a study that included both healthy and lung adenocarcinoma patients, the same cancer type as our cfNano samples. In our new analysis of complete cell type composition, the two are in excellent agreement (Figure 1B-C). This provides an important negative baseline for cell type analysis in healthy samples, which agree between the different platforms and with what has been described previously in the literature.

We also added three new healthy Nanopore samples sequenced at our second source site (Hebrew University in Israel), and showed that cell type composition and all other methylation and fragmentomic features were similar to our original samples (sequenced at ISPRO in Italy).

2)	Do they see any difference in the accuracy or utility of the mC sites if they use a different base caller, like Megalodon? Making the claims on these sites is likely somewhat dependent on the base caller they used (DeepSignal), and so this should be teased out, validated, and quantified.

We now compare methylation using the original pipeline (DeepSignal) with the newest available pipeline (Megalodon using the Remora model). The majority of individual CpG methylation calls are identical (Supplementary Figure 6A), and we show in side-by-side analyses that deconvolution (Supplementary Figure 6B-C), TFBS demethylation (Supplementary Figure 7C), and PMD hypomethylation (Supplementary Figure 7D-F) are nearly identical. Small differences in deconvolution tended to favor the Megalodon pipeline (which also called significantly more CpGs), so we used that one for the main figures and put DeepSignal versions in Supplementary Figures 6-7. This concordance is reassuring, but not surprising given that several recent benchmarking studies showed that the top methylation callers (including these two) have converged in accuracy.

Z. W.-S. Yuen, A. Srivastava, R. Daniel, D. McNevin, C. Jack, E. Eyras, Systematic benchmarking of tools for CpG methylation detection from nanopore sequencing. Nat. Commun. 12, 3438 (2021).

Y. Liu, W. Rosikiewicz, Z. Pan, N. Jillette, P. Wang, A. Taghbalout, J. Foox, C. Mason, M. Carroll, A. Cheng, S. Li, DNA methylation-calling tools for Oxford Nanopore sequencing: a survey and human epigenome-wide evaluation. Genome Biol. 22, 295 (2021).

For fragmentomics, we also added side-by-side comparisons of two Nanopore base calling pipelines, one that was available originally when the sequences were generated in 2019, vs. the newest one available in 2022. There were only very subtle differences in fragmentomic properties, but the newer pipeline produced results that were slightly more similar to matched Illumina WGS samples (Figure 4C,E,G,H,J).

3)	The authors noted that "ATAC-seq open chromatin regions are almost universally demethylated in cancer," and they also saw this in their samples, but did they observe the opposite trend in closed sites across the genome as well?

In general, while there are many sites that are “cell-type specific open” (open in one cell type and closed in all others), there are very few sites that are “cell-type specific closed”. The scATAC-seq atlas that we use called no such “cell-type specific closed” sites. However, to test the hypothesis and provide a negative control, we added a second set of cell-type specific sites for a cell type not expected in the blood of either healthy or lung cancer individuals (adrenocortical cells). These showed no demethylation in either healthy plasma or lung cancer plasma, from either our cfNano samples or the new Illumina WGBS cfDNA dataset that includes lung cancer patients (Supplementary Figure 7B).

We considered adding this negative control data to the main figure, but decided to include it in the supplemental figure instead, especially given Reviewer #2’s concern about the inclusion of non-essential analyses in the main figures.

4)	They found that the lung specificity of the NKX2-1 sites was quite distinct, which makes sense given it is a transcription factor in lung pneumocytes. Are there other lung-specific signatures they could use to validate this trend?

Our “two-component” lung cell deconvolution is based on regions that differ between normal lung epithelia and normal blood cells in healthy plasma. In order to check whether these regions were primarily derived from NKX2-1 binding sites, we overlapped the two CpGs sets, which did not identify even a single overlapping CpG. It is maybe not so surprising since the HM450k array used for the deconvolution was designed in 2010: it is represented mostly by promoters and did not include a large number of enhancer regions such as NKX2-1. It is nevertheless likely that the deconvolution CpGs contain other lung-specific signatures, but we could not find other reference datasets from lung epithelial cells to characterize these, and leave that for future work. Unfortunately we could not find any other transcription factor binding site with a specificity similar to that of NKX2-1.

5)	Related to #4, do the authors find any novel sites that might be differentiating between the lung tissue and normal? Knowing they are underpowered, this would just be exploratory, but it would show the potential of the method to discover new markers and regulatory regions.

Indeed, it would be a sensible undertaking but, as the reviewer implies, not only the number of samples is too small, but the current level of genomic coverage makes it extremely underpowered for this kind of discovery. We randomly cover only about 20% of CpGs per sample (about 5 million). This means that any two samples only overlap by approximately 20%*20%=4% of all CpGs (about 1 million). We don’t believe greater coverage would be required, but greater sample number definitely would, possibly a reasonably sized study of 50 or so cfNano samples from a particular tumor type. We added this idea to the Discussion section on lines 364-366.

6)	The default software for ONT does not allow fragments below 200nt; did the authors need to adjust the run-time software to account for this, or make any other modifications to the ONT flowcell processing?

The runtime software available at the time of our sequencing allowed for 100nt fragments but not below. Due to interest in our preprint and other short-read applications, Oxford Nanopore has recently announced that changes in their runtime software will soon allow efficient recovery of short fragments ([link](https://nanoporetech.com/about-us/news/oxford-nanopore-releases-short-fragment-mode-new-tool-real-time-sequencing-short?utm_campaign)). Unfortunately, this is a change that has to be applied at run-time, and can not be used post-hoc to recover short fragments from older sequencing runs - they have already been lost. We used the standard runtime software for all our analyses, and have added this to our Methods section.

7)	The authors mention that "higher read depth will be necessary to detect nucleosomal fragmentation patterns from cell-type specific components of cfDNA," but some down-sampling or estimate of this would be good to see, and in general, to help guide future experiments in this direction. Their conclusions recommend a mean of 6.4M reads, but it would be nice to know how low we can go (2.6M seems a little low, for example in BC10), and if 3-4M reads might be enough. A deconvolution accuracy with incrementing sets of 1M reads would be very illustrative and is recommended.

Even the 2-15M reads sequenced here was insufficient to detect nucleosome positioning at NKX2-1 sites, where the nucleosome positioning would only occur on a fraction of the fragments (those derived from cancer cells). We removed our speculation about what read depth would be necessary, since it is very unclear from other published work. The gold standard 2019 paper from Dennis Lo (doi:10.1101/gr.242719.118) used cancer plasma samples with a median depth of 64 million uniquely mapped fragments. A recent preprint with a new bioinformatic approach, used cancer samples with a median depth of 45,000,000 fragments (<https://doi.org/10.1101/2021.04.14.439883>). Downsampling from our current coverage of 6-15M reads would be going the wrong direction. In our new text, we suggest that perhaps improved bioinformatic normalization techniques could help, but this is still speculation. The main point is that the nucleosome signal appears to be very similar to that of matched Illumina WGS samples, based on the CTCF analysis. So whatever signal is there to find in Illumina data, it is also there in cfNano. Our results suggest that methylation is a cleaner signal, since we can detect that at NKX2-1 sites but not nucleosome positioning. We improved our discussion of this on lines 229-236.

8)	There is one additional benchmarking study they should reference that supports their claims and also observed that the motif differences they observe between Illumina and ONT could be a function of the ONT base caller: Foox et al., Genome Biology, 2021. Also, a table of their other predicted motifs would be useful.

This comment prompted us to re-analyze all our cfNano data with the newest version of the Nanopore base caller, which uses the “High Accuracy Calling” Model, and compare it to the Fast calling model we used with the original 2019 version of the base caller. Interestingly, this new base calling made the motifs more similar to Illumina , suggesting that some errors may have been improved.

We added new plots of these data as Figure 4G-H to show that the two platforms mostly agree but still have small differences. This is a bit complicated though, because the errors most likely come from adapter trimming and possible misalignments at the ends of the reads rather than errors in the 4-mers themselves (because we use the reference genome to extract the 4-mer sequences). This new analysis changed our perspective on end motifs, suggesting that they are quite susceptible to sequencing platform and even batch effects (they also differed between our original batch of “ISPRO” samples and our newly sequenced “HU” samples). So we added a significant amount of discussion of this in lines 309-319 and 337-347. End motifs are a relatively new feature first described in 2020, so we feel this is a useful discussion and analysis. We appreciate your prompting to look into this issue further, and also cited the excellent article you pointed out.

We also added a new Supplementary Table 3 with cancer vs. normal comparisons for all 256 motifs, and a discussion of this on lines 305-306. We found additional 16 motifs that were as significant as CCCA, although none survived FDR adjustment and so will have to be validated in larger studies.

Reviewer #2: In the article entitled "Detecting cell-of-origin and cancer-specific features of cell-free DNA with Nanopore sequencing" Katsman et al describe an approach based on Nanopore sequencing for analyzing epigenetic features of cell-free DNA (cfDNA) in plasma. In particular, using nanopore sequencing could allow to by-pass the degradation linked to bisulfite conversion to analysis 5mC in minute amounts of cfDNA.

The authors major claims, that Nanopore sequencing can retrieve cell of origin and cancer specific features on cell-free DNA, are only partially supported by the results presented.

1. The sensitivity and accuracy for determining the cell of origin of cfDNA is not concretely determined. The authors are not using any form of positive control to determine the accuracy for determining the cell of origin of cfDNA in plasma (e.g. what fraction of white blood cell is detected using Nanopore, how does this compare to a control, is it better or worse than Illumina based approaches?). Viewing this is a feasibility study, I am not convinced comparison with database will be enough to accurately determine such populations.

We have now added a more comprehensive version of the deconvolution analysis which resolves all cell types covered by the methylation atlas (Figure 1A-C and Supplementary Figures 1-3). As expected from earlier work, we find the bulk of healthy plasma to be dominated by monocytes, neutrophils/granulocytes, megakaryocytes, and hepatocytes. There is a new dataset available of deep (80x) Illumina WGBS sequencing of healthy plasma cfDNA samples (Fox et al. eLife 2021), and we show that downsampling these data to the coverage level of our nanopore samples (0.2x) introduces only small changes, and (2) yields cell type proportions similar to our healthy nanopore samples. We performed the same deconvolution on samples from another new WGBS study which contains both healthy plasma vs. lung adenocarcinoma (LuAd) plasma samples. This was extremely fortuitous since our cancer samples are also LuAd. These samples showed cell compositions very similar to our cfNano samples, both for the healthy and for the lung cancer samples (Figure 1A-C). While there is a significant degree of variability in normal cell types even with the 80X WGBS coverage (presumably biological based on past studies), we did not find significant differences between the two WGBS datasets and our cfNano dataset.

We also sequenced 3 additional healthy samples at a second sequencing site (Hebrew University). Despite lower sequencing yield (two of the three did not pass the 0.2x threshold), they also gave similar cell type proportions to the other cfNano and Illumina WGBS samples.

Our ability to define accuracy and sensitivity will require a larger cohort. However, we believe that our new analyses and comparisons to Illumina datasets are overwhelmingly positive and nothing so far indicates major concerns for the Nanopore technology. Our downsampling experiments did find that a small amount of noise was introduced at the 0.2x level, and we discuss this openly on lines 348-358. We believe that more read will be required, especially for sensitive detection, but we also believe that improve whole genome methylation atlases can improve the resolution significantly (we use an array-based atlas, which only includes 10-15% of cell type marker regions).

2. The size of the cohort of matched Illumina and ONT samples is small. Even if this is a feasibility study, the manuscript results would be more convincing with a larger cohort of matched Illumina-Nanopore cases.

Unfortunately, we are not currently able to collect additional cancer samples at this time. As described above, we have added a substantial amount of external Illumina WGBS data to validate the methylation results. While this is not as good as matched Illumina WGBS samples, we believe they strongly suggest good performance. And, as described in the response to question 3 below, we have added more systematic side-by-side fragmentation analysis of the four matched WGS cancer samples that we do have (Figure 4B-J), and we believe this led to interesting and important insights. We also included a new side-by-side analysis of CNA performance of cfNano and Illumina WGS on the matched samples (Figure 1E). While the number of samples is too small to be conclusive, cfNano samples were more similar to the high-depth Illumina samples than the Illumina samples themselves were when downsampled to the same depth as the cfNano samples (Figure 1E vs. Supplementary Figure 5A).

3. The results in figure 2 are not supporting the claim that Nanopore is preserving cfDNA fragmentation features. Even if Illumina and Nanopore data are vaguely compared, a much more throughout point-by-point quantitative comparison per matched samples on various fragmentomic features is needed to draw such strong conclusions. Is the signal maintained when coverage is reduced?

While there is a limit to the conclusions we can draw from four matched samples, we have now added systematic comparisons for each fragmentomic feature. Importantly, we are not relying *only* on these head to head comparisons, since we are only showing known cancer-associated features that were originally discovered in illumina-based studies. For instance, for the short mononucleosome analysis, we use the same calculation of fragment length ratio as was used in two of the landmark papers (Mouliere 2018 and Cristiano 2019, Figure 4B-C). For the 4-mer end motifs, we investigate the cancer-associated motif (CCCA) that was described by the most prominent papers in the field (Jiang et al. 2020 and Chan et al. 2020, Figure 4G-J). We rank the other motifs and plot frequencies in exactly the same format as in their newest paper (Chan et al. 2020, Figure 4F).

In addition to systematic fragmentomic comparisons of the four matched cfNano/Illumina samples, we also make several new comparisons across cfNano data. First, we compare batch effects between our original healthy samples and three new healthy samples, sequenced almost 2 years later (HU samples, shown in all panels of the fragmentomic Figures 3 and 4). We also do side-by-side comparisons of all original samples called with the original 2019 base calling model vs. the newer improved model (Figure 4C, E, G, H, J).

Based on the more rigorous fragmentomic analysis, we were able to gain a better understanding of how different fragmentomic features behave across platforms and batches. We were able to conclude that fragment lengths are likely robust enough to be compared across Nanopore batches and also probably across platforms. Dinucleosome lengths seemed much more robust than mononucleosome lengths both with respect to cancer-specificity as well as platform concordance. In contrast, we found that end motifs are not robust across platforms or even different batches on the same platform. This is likely due to the fact that end alignments and adapter trimming are particularly sensitive to sequencing errors and bioinformatic pipelines, as well as library construction. So we added a significant amount of discussion of this in lines 309-319 and 332-347. End motifs are a relatively new feature first described in 2020, so we feel this is a useful discussion and analysis.

Notwithstanding the caveats above, when comparing within a batch the major fragmentomic features reliably reflected what was discovered earlier using Illumina - i.e. short mononucleosomes and dinucleosomes are more abundant in cancer, and the CCCA motif is less abundant in cancer. We also performed downsampling of all of these features to 2M reads (Figure 3C, Supplementary Figures 9E-H, Supplementary Figures 10C-D), which had no observable effect.

4. One figure has 14 panels, the other one 7 panels. I think the authors should do an effort to be as concise and specific as possible and move to supplementary materials and anecdotal or minimally processed results. This could increase the clarity and potential impact of the manuscript.

We added a significant number of new analyses based on the reviewer suggestions, but we have kept ourselves to a total of 23 main figure panels total (spread across 4 main figures). The majority of Figure 1 is new, as is the majority of Figure 4. We have tried to limit the main figures to the most essential information.

5. In general, the claimed added value of Nanopore sequencing versus Illumina seem anecdotal viewing the potential negative sides (or at least limitations, which should be discussed more in depth). In particular the sensitivity for detecting scarce signal might be lower than Illumina and potential for multiplexing (and thus cost/sample) higher. I agree that the potential of Nanopore for providing quickly results render the technology conceptually superior to Illumina for emergency medicine, but some direction of applications (or even better, preliminary data) could enhance the work.

In retrospect, we agree that our first submission was not as balanced as it should have been. We have added counterpoints in both the Introduction (lines 50-54) and Discussion (lines 359-374). The most important strength of Illumina is probably the ability to do targeted methylation sequencing, which is not a strong point for ONT. Base substitution and indel error rates are also still a lot better with Illumina, and we point this out as a consideration. We also address the cost issue, and the limited throughput of the MinION platform (both are meant to be addressed by PromethION, which ONT is dropping the price of drastically).

We list more specific applications in emergency medicine, including new Nanopore methylation papers in intraoperative tumor classification during brain cancer surgery, and major Illumina-based methylation studies in myocardial infection, sepsis, COVID-19 (lines 386-390).

The most important strength of Nanopore (even more than realtime) is that it can yield multiple cancer-associated features (methylation, CNA, fragmentomic) in a very simple native DNA sequencing assay, without chemical or enzymatic processing to read out methylation. The leading Illumina-based technology, WGBS, gives inconsistent results with low sample input, due to the loss of DNA associated with bisulfite treatment. This makes it a challenge for clinical applications.

I have also the following comments:

-	Line 34: Conventional ONT sequencing is vague and misleading, as the reader most certainly understands amplicon-based sequencing. Please clarify that you used native DNA sequencing.

We removed all instances of the word conventional. We refer to it only as “native” DNA sequencing

-	Lines 48-50: The authors write about detecting unusual cell types in cfDNA, but this is out of context. Please add one line before this to highlight the expected cell type composition detectable from cfDNA.

We have removed this, since we now show in detail the expected cell types with our new healthy cell type deconvolution results.

-	Line 78: Please mention the detection limit of ichorCNA explicitly in numbers.

This is now described in the Methods section, lines 704-706. We call it undetectable if less than 15% of the genome is covered in CNAs.

-	Lines 88-91: Please add the mean and SD methylation levels for controls and cancer samples.

We have added that now in lines 199-201 corresponding to the new Figure 2C. We also computed a one-sided wilcoxon value for all cancer bins vs. all healthy bins (p<2.2e-16).

-	Lines 190-195: Please add the mean and SD methylation levels for controls and cancer samples.

We have added that now in line 261-263 corresponding to the new Figure 4B. We have also added the same for the other panels that used t-tests, Figure panels 4D and 4I.

-	Lines 210-211. Please say specifically the direction of the difference for CCAA. Also mention the relatively high error rate of the ONT technology, and that you filtered soft-clipping.

The direction of difference of CCCA is now made clear on line 294-298 and in Figure 4I, and we also give the mean and SD to make it more clear. We now have a fuller discussion of the pitfalls of end motif analysis, and mention explicitly that we exclude soft-clipped ends on line 315.

Importantly, we even see differences between our two cfNano batches, which were both processed with the same bioinformatic pipeline (except for the initial runtime control and data acquisition, which was done on Nanopore systems almost 2 years apart). This suggests that, even if the overall results hold across time and batches, in the future proper controls need to be developed to compare datasets sequenced at different times (this is almost certainly true for Illumina WGS as well, since this feature was only discovered in the past 2 years and to our knowledge has never been combined across different batches/datasets). We feel we made this point sufficiently in the Discussion on lines 337-347, but please give us additional feedback if it’s not strong enough.

-	Materials and methods, lines 153-156: Please clarify if you extracted the genomic sequence or the actual read sequence for the analysis? If the latter, did you take any measures to filter reads with unmapped single, double or triple bases in the 4-mer? If the latter, please provide a supplementary figure showing the proportion of reads with soft-clipping, unmapped bases and fully mapping bases in their 5' 4-mers.

All 4-mer sequences were extracted from the reference genome. We have now added that detail to the Methods on lines 835-836, and the Discussion section on line 338-340. We did that precisely to avoid the kind of problems with Nanopore base calling errors that you suggest (this is the same reason we removed soft-clipped reads).

-	Figure 2C: please use colorblind friendly colors in the heatmap.

We have changed that it to the Tol version of rainbow (https://cran.r-project.org/web/packages/khroma/vignettes/tol.html#rainbow)

**Second round of review**

**Reviewer 2**

Technically, I have not major issue with the revised version of this manuscript. The authors have nicely enhanced the quality of their manuscript, even if they have not followed completely my recommendations for improvement.
I am however sceptical about the good correlation between Illumina and ONT results, in particular for the fragmentomics analysis, and wonder how much this could be biased by the ONT protocol chosen (e.g. whether it includes size selection with increased beads ratio or not). Recent results using PacBio technologies seems to confirm that a large fraction of long cfDNA fragments are missed using Illumina sequencing. I suggest the authors to add a word of caution in the discussion, as the use of different ONT kits (or pre-analytical conditions) might potentially change their observations in the future.

I have also the following minor comments:
Abstract: I would remove the sentence “especially for real-time medical applications”. I feel this claim, even if possible on the long term, is misleading in the abstract as the authors do not demonstrate it. This is more an element for discussion in my opinion.
Line 101: “LuAd” is repeated.
Line 114: The author claims the fraction of cancer cells can be estimated in cfDNA. This wording can be misleading as ichorCNA can detect signals only from cells with copy number alterations only. Please consider rewording.
Lines 131-132: Our in-house results with ichorCNA show that the TF estimation can be way off compared to what would be expected by looking at the segmentation plot. BC10’s segmentation plot shows extensive SCNAs suggesting a much higher TF than the 30% estimated by ichorCNA. Our experience is, that changing the panel of normals could result in a more realistic estimation. Did the authors generated their own panel of normals or used the one provided by ichorCNA. Please specify this in the Materials and methods section.
Line 600-601: In the Methods section, the demultiplexing parameters for the Fast model are missing. Please add them if applicable.
Line 646: typo: “profide”

**Authors Response**

**Point-by-point responses to the reviewers’ comments:**

Reviewer #2: Technically, I have not major issue with the revised version of this manuscript. The authors have nicely enhanced the quality of their manuscript, even if they have not followed completely my recommendations for improvement.

We appreciate these comments. We did all we could with the constraint that we did not have access to additional cancer samples.

I am however sceptical about the good correlation between Illumina and ONT results, in particular for the fragmentomics analysis, and wonder how much this could be biased by the ONT protocol chosen (e.g. whether it includes size selection with beads or not). Recent results using PacBio technologies seems to confirm that a large fraction of long cfDNA fragments are missed using Illumina sequencing. I suggest the authors to add a word of caution in the discussion, as the use of different ONT kits (or pre-analytical conditions) might potentially change their observations in the future.

This is a good point and worth pointing out, especially given the dynamic nature of ONT. We have added a new final sentence to the 2nd paragraph of the Discussion, urging caution.

I have also the following minor comments: Abstract: I would remove the sentence "especially for real-time medical applications". I feel this claim, even if possible on the long term, is misleading in the abstract as the authors do not demonstrate it. This is more an element for discussion in my opinion.

In retrospect, we agree that the placement of that phrase “real-time sequencing” at the end of the Abstract was inappropriate, because it could lead to the impression that we actually showed data about that (which the reviewer correctly points out we did not). However, we think it’s one of the most important reasons to consider using the Nanopore technology, so we kept it but moved it to the more appropriate place in the Abstract, which is the introductory sentence.

Old text: “The Oxford Nanopore (ONT) platform provides portable and rapid genome sequencing, and its ability to natively profile DNA methylation without complex sample processing is attractive for clinical sequencing”

New text: “The Oxford Nanopore (ONT) platform provides portable and rapid genome sequencing, and its ability to natively profile DNA methylation without complex sample processing is attractive for point-of-care real-time sequencing” We hope the editor and reviewer will agree with this change.

Line 101: "LuAd" is repeated. Fixed Line 114: The author claims the fraction of cancer cells can be estimated in cfDNA. This wording can be misleading as ichorCNA can detect signals only from cells with copy number alterations only. Please consider rewording. Good point.

We changed “for cancers that contain a sufficient degree of aneuploidy” to “for cancer cells that contain a sufficient degree of aneuploidy”.

Lines 131-132: Our in-house results with ichorCNA show that the TF estimation can be way off compared to what would be expected by looking at the segmentation plot. BC10's segmentation plot shows extensive SCNAs suggesting a much higher TF than the 30% estimated by ichorCNA. Our experience is, that changing the panel of normals could result in a more realistic estimation. Did the authors generated their own panel of normals or used the one provided by ichorCNA. Please specify this in the Materials and methods section.

We used the built in panel of normals (default ichorCNA settings). We did not use our own panel of normals because the only normals we have are the normals included in this study, and we specifically did not want to bias our comparison of normal to cancer in any way. However, we think it was unlikely that the tumor purity was strongly affected in the case of BC10, or in the other cases, because they were nearly identical when compared to deep Illumina WGS (Figure 1E).

Line 600-601: In the Methods section, the demultiplexing parameters for the Fast model are missing. Please add them if applicable.

Thanks for reading so carefully. We added the specific parameters now. We also changed the name from “fast model” to “high-accuracy real-time” mode, which was the official name of the model at that time, and added a few extra details to this section of the Methods to make things clearer overall.

Line 646: typo: "profide"

Thank you. We actually had “profide a simple bedgrpah”, so we fixed the “bedgrpah” as well :)
